# Tinnitus: animal models and findings in humans

**DOI:** 10.1007/s00441-014-1992-8

**Published:** 2014-09-30

**Authors:** Jos J. Eggermont, Larry E. Roberts

**Affiliations:** 1 Department of Physiology and Pharmacology, Hotchkiss Brain Institute, and Department of Psychology, University of Calgary, 2500 University Drive N.W, Calgary, AB Canada; 2 Department of Psychology, Neuroscience, and Behaviour McMaster University, Hamilton, ON Canada

**Keywords:** Tinnitus, Hyperacusis, Deafferentation, Cochlear neuropathy, Auditory system

## Abstract

Chronic tinnitus (ringing of the ears) is a medically untreatable condition that reduces quality of life for millions of individuals worldwide. Most cases are associated with hearing loss that may be detected by the audiogram or by more sensitive measures. Converging evidence from animal models and studies of human tinnitus sufferers indicates that, while cochlear damage is a trigger, most cases of tinnitus are not generated by irritative processes persisting in the cochlea but by changes that take place in central auditory pathways when auditory neurons lose their input from the ear. Forms of neural plasticity underlie these neural changes, which include increased spontaneous activity and neural gain in deafferented central auditory structures, increased synchronous activity in these structures, alterations in the tonotopic organization of auditory cortex, and changes in network behavior in nonauditory brain regions detected by functional imaging of individuals with tinnitus and corroborated by animal investigations. Research on the molecular mechanisms that underlie neural changes in tinnitus is in its infancy and represents a frontier for investigation.

## Introduction

Tinnitus is the conscious perception of sound heard in the absence of physical sound sources external or internal to the body. Sound perceived from physical sound sources inside the body such as blood flow and middle ear muscle twitching is generally called “objective tinnitus”; in this paper we will only consider subjective tinnitus and just call it “tinnitus”. Tinnitus occurs in children as well as in the elderly, in war veterans and factory workers, and in classical musicians, rock stars and disc jockeys. About 15 % of adults experience tinnitus. In a recent study of 40–69 year olds in the UK (*n* = 164,770), 16.9 % reported tinnitus (Dawes et al. 2014). Tinnitus is generally ignited by hearing loss, and very often by noise exposure. Most chronic tinnitus has a central origin: that is, tinnitus is in the brain and not in the ear; it is only referred to the ear. An example illustrating this is found in people with one-sided deafness, who often experience tinnitus referred to the deaf ear, yet the tinnitus often subsides when that ear is stimulated via a cochlear implant (Van de Heyning et al. 2008). The localization of tinnitus to one or both ears is thus attributable to a phantom sensation not unlike that related to sensations or pain experienced after losing a limb. Itching or pain in a no-longer-existing part of the body is truly annoying and so is tinnitus. Although about 15 % of adults experience tinnitus, in 1–2 % of adults the tinnitus is sufficiently persistent and distressing that quality of life is reduced and medical help is sought. For the large majority of cases of persistent tinnitus, there are at present no effective medical treatments for the tinnitus sound, although one’s reaction to having tinnitus can be modified (Hoare et al. 2014).

Electrophysiological and functional imaging measurements in humans and animals suggest that increased neural synchrony, tonotopic map reorganization, and increased spontaneous firing rates (SFR) in the auditory system are potential neural correlates of tinnitus (Eggermont and Roberts 2004; Roberts et al. 2010). Tinnitus is likely the result of maladaptive plasticity of the central nervous system. The central nervous system aims to restore its normal evoked neural activity levels that had been lowered in the frequency range of the hearing loss. This is done by increasing the synaptic efficacy (or gain) in central auditory neurons (Turrigiano 1999). But this gain change also affects the SFR, which occurs in the absence of a physical sound source, and thus the SFR will also increase. This is interpreted as sound and called tinnitus. A puzzling aspect is that only 30 % of people with hearing loss experience tinnitus; conversely, it has been estimated that 15 % of individuals assessed for chronic tinnitus have clinically normal audiograms. These results suggest either that there are other extra-auditory nervous system aspects that promote or allow the perception of increased SFR and neural synchrony in auditory pathways as tinnitus, or that the neural changes in auditory pathways generating tinnitus depend on cochlear pathologies not detected by the audiogram. Clinical audiograms rarely measure thresholds for frequencies >8 kHz. It is worth noting here that clinical normal hearing allows up to 20 dB loss at frequencies between 125 and 8,000 Hz. As we will indicate later even modest differences within this 20-dB range may have considerable impact on particularly spontaneous firing rates in central brain structures, and differences within this range may relate to the presence or absence of tinnitus in human subjects.

About two decades ago, research on tinnitus was focused on animal models; however, the increasing availability of neuroimaging techniques resulted in a surge of human studies. Not only functional magnetic resonance imaging (fMRI) but also electroencephalography (EEG)- and magnetoencephalography (MEG)-based studies in tinnitus patients took off. Some of these studies were comparable with the animal ones as they focused on the auditory system, particularly the auditory cortex, with an emphasis on spontaneous activity, neural synchrony and tonotopic mapping (Roberts et al. 2010). Another group of studies ventured beyond the auditory cortex and surveyed changes in neural connectivity between brain regions in patients with tinnitus (Weisz et al. 2007; De Ridder et al. 2011). These studies suggested that the auditory system is largely irrelevant for understanding the impact of tinnitus on the quality of life. One wonders if this means that animal research into the basic mechanisms of tinnitus should be abandoned. Consequently, animal models of tinnitus are at a crossroads: clinical interest is in patients’ suffering, but alleviation of this suffering does not abolish tinnitus. Greater basic understanding of the generation of tinnitus, an auditory percept, certainly needs more and better animal models.

It is the purpose of this review to describe how we got to our current understanding of tinnitus based on a comparison of animal models with compatible data from human studies. As noted above, most but not all cases of chronic tinnitus are observed in individuals who have experienced some degree of hearing loss as defined by standard clinical audiometry (thresholds <20 dB HL to 8 kHz). We therefore begin in “Hearing loss, tinnitus, and hyperacusis” with a brief preliminary overview of the role of hearing loss in tinnitus and of the neural changes that take place in auditory pathways when hearing loss is present. These topics are elaborated in more detail in subsequent sections beginning with a review of “Animal studies” and “Human studies”. Within these sections, we consider the methods that have been used to investigate tinnitus mechanisms (principally physiological experiments in animals and functional brain imaging studies in humans) as well as the main findings emerging from this work. In “Cochlear pathology revisited: animals and humans”, we discuss recent intersections between animal and human research that may improve our understanding of the role of cochlear mechanisms in initiating the central neural changes that underlie tinnitus. Understanding how hearing loss is involved has implications for the assessment and treatment of hearing disorders and for charting future directions in basic and translational research. A brief summary and look ahead is presented in “Summary, conclusions, and limitations”.

## Hearing loss, tinnitus, and hyperacusis

### Tinnitus

**Fig 1:**
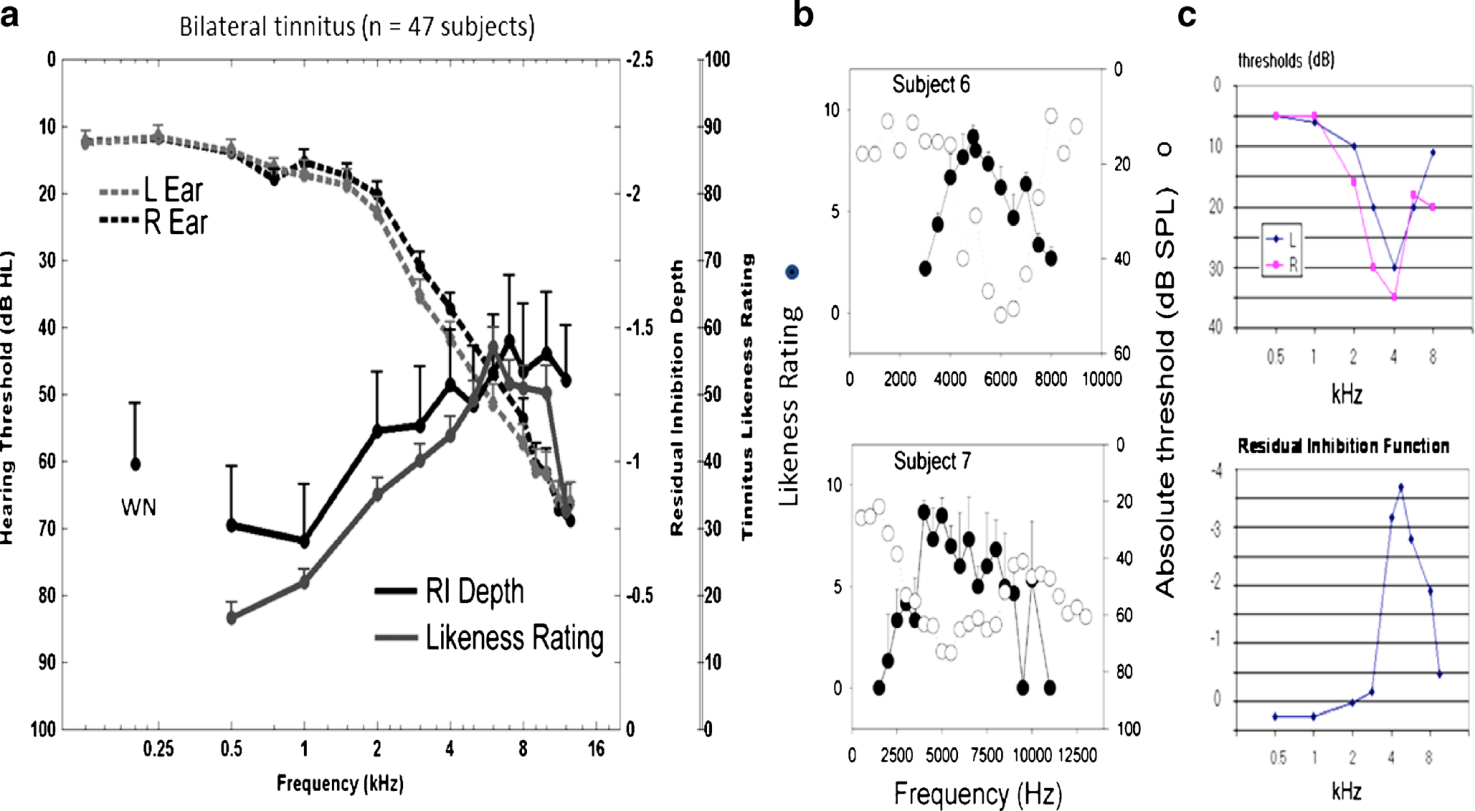
Psychoacoustic properties of tinnitus. **a** Sound frequencies judged to resemble tinnitus (*Likeness Rating*) and the center frequency of band pass maskers giving optimal forward suppression of tinnitus (residual Inhibition,* RI Depth*) track the region of audiometric threshold shift (from Roberts et al. 2008). A likeness rating of 40 denotes a sound beginning to resemble tinnitus. Sound thresholds (*broken lines*) are considered normal when ≤ 20 dB HL.* WN* RI depth after a white noise masker. **b**, **c** When audiometric notches are present, Likeness Ratings (**b**) and RI Depth (**c**) follow this principle. Two individual subjects are shown in (**b**) from Noreña et al. (2002) and one subject in (**c**) from Roberts (2007). During RI in (**a**) and (**c**, *lower panel*), tinnitus elimination corresponds to an RI depth of −5.0

Hearing loss, resulting for instance from exposure to loud noise, is considered an important risk factor for developing tinnitus. Consequently, a history of recreational, occupational, and firearm noise exposure may all be associated with increased likelihood of acquiring tinnitus. The relationship between noise exposure and tinnitus, however, differs depending on the presence or absence of hearing impairment. Occupational noise exposure is more likely to correlate with significant tinnitus in participants with hearing impairment, while leisure-time noise exposure is more associated with increased occurrence of significant tinnitus in participants without audiometric (frequencies ≤8 kHz) hearing loss (Eggermont 2012, pp. 21–24). While this dissociation could reflect early occurring cochlear changes to which the audiogram is not sensitive (a topic discussed in “Cochlear pathology revisited: animals and humans”), it is clear that, when audiometric hearing loss is present, the frequencies reported by patients to correspond to their tinnitus are in the frequency region of threshold shift in the audiogram (Noreña et al. 2002; Roberts et al. 2008; see Fig. 1) with the dominant pitch most commonly reported for NIHL-induced tinnitus matching that of a 3-kHz tone (Penner 1980). Whether the dominant pitch of tinnitus is at the audiometric edge of hearing loss or well within the hearing loss region is still debated. The prediction of overrepresentation of edge frequencies in tonotopic maps after noise trauma, implying that tinnitus pitch would match the edge frequencies (Rauschecker 1999), could not be confirmed (Roberts et al. 2008; Pan et al. 2009). Moore and Vinay (2010) assessed whether this failure might be related to octave errors in pitch matching. Following the training of participants to avoid these errors, the mean pitch matches were close to the values of the edge frequency, with a correlation coefficient of 0.94. In contrast, Schecklmann et al. (2012) confirmed a relationship between tinnitus pitch and maximum hearing loss but not to the edge frequency, suggesting to them that tinnitus is rather a fill-in-phenomenon resulting from homeostatic mechanisms (Roberts et al. 2008) rather than a result of contrast enhancement and the audiometric edge consequent deficient lateral inhibition (Llinás et al. 2005). While these disparate findings could reflect that even in tonal tinnitus a band of frequencies may be present, they concur regardless that tinnitus frequencies are related to hearing loss. Interestingly, narrow-band maskers giving a brief post-masking suppression of tinnitus (called “residual inhibition” in the tinnitus literature, RI) do so most effectively when the center frequency of the masker is also in the hearing loss region (see Fig. 1). Overall, these results suggest that neurons tuned to frequencies in the hearing loss region do generate tinnitus, and stopping what they do suppresses it.

Phantom auditory sensations can be induced in normal hearing listeners when they experience auditory deprivation such as confinement in an anechoic, sound insulated, chamber (Heller and Bergman 1953; Tucker et al. 2005; Del Bo et al. 2008). Schaette et al. (2012) described the emergence of phantom sounds after continuous use of an earplug. Eighteen healthy volunteers with normal hearing wore a silicone earplug continuously in one ear for 7 days. The attenuation provided by the earplugs simulated a mild high-frequency hearing loss; mean threshold increased from **<**10 dB at 0.25 kHz to **>**30 dB at 3 and 4 kHz. Fourteen out of 18 participants reported phantom sounds during earplug use. Eleven participants presented with stable phantom sounds on day 7 and underwent tinnitus spectrum characterization with the earplug still in place. The spectra showed that the phantom sounds were perceived predominantly as high-pitched, corresponding to the frequency range most affected by the earplug. In all cases, the auditory phantom disappeared when the earplug was removed, according to the authors indicating a causal relationship between auditory deprivation and phantom sounds. However, we note that the findings may also be interpreted as the absence of environmental masking being the cause of the tinnitus percept.

### Hyperacusis

Hyperacusis, an increased sensitivity to sound at levels that would normally not be of discomfort to an individual, has been associated with exposure to sound and is often reported in people with hearing loss. Hyperacusis, as defined, is likely the consequence of homeostatic adjustments in synaptic activity (Eggermont 2012). In a study by Schecklmann et al. (2014), hyperacusis was defined by the question: do sounds cause you pain or physical discomfort? When patients who answered this question with “yes” were contrasted with “no” responders, 935 (55 %) out of 1,713 patients were characterized as hyperacusis patients. The high prevalence of hyperacusis in tinnitus subjects suggests that both symptoms have a common origin, and that they may result from an increase of central gain attributable to sensory deafferentation. More specifically, tinnitus and hyperacusis could result from an increase of spontaneous and stimulus-induced activity, respectively. One prediction of this hypothesis is that loudness sensitivity should be increased in tinnitus compared with non-tinnitus subjects. Hébert et al. (2013) tested this prediction by examining the loudness functions in tinnitus ears (*n* = 124) compared with non-tinnitus human ears (*n* = 106). Tinnitus and non-tinnitus ears were carefully matched for hearing loss (thresholds ≤15 dB HL). The results show that loudness sensitivity is indeed enhanced in tinnitus subjects compared with non-tinnitus subjects, including subjects with normal audiograms. How far these results are generalizable to the 45 % of tinnitus sufferers who do not volunteer reports of increased sensitivity to environmental sounds (Schecklmann et al. 2014) is not known. The possibility remains that changes in central gain responsible for hyperacusis while setting the stage for tinnitus may not be sufficient for its occurrence. Additional mechanisms such as aberrant neural synchrony in central auditory structures or access of tinnitus-related neural activity to nonauditory brain regions responsible for consciousness may be needed to perceive the sound of tinnitus (De Ridder et al. 2011; Roberts et al. 2013).

## Animal studies

Given the aforementioned results in humans, it is not surprising that procedures that induce hearing loss have been used widely to investigate the basis of tinnitus in animals. Initially, a main goal was to identify the neural changes that are induced by hearing loss and are therefore possible neural codes for tinnitus and hyperacusis. More recently, behavioral assessments of tinnitus have been devised with the aim of identifying which neural changes are associated specifically with tinnitus or hyperacusis and not with hearing impairment alone.

### Salicylate versus noise exposure

Ingestion of large doses of salicylate in humans causes tinnitus, hearing loss, and changed sound perception. These symptoms develop over the initial days of use but may then level off, fluctuate or decrease, and are reversible within a few days of stopping the drug use. After exposure of humans to loud traumatic sounds tinnitus develops almost immediately. After traumatic noise, prolonged exposure to occupational or recreational noise, or following slowly acquired hearing loss during aging, tinnitus may over time develop from an intermittent presence to chronic. Chronic tinnitus likely reflects an intrinsic central source. Table 1 shows a comprehensive account of changes following chronic salicylate and noise exposure for auditory structures ranging from the auditory nerve to auditory cortex. The noise-exposure data are shown in bold. Upward-facing arrows indicate increases, downward-facing ones reductions, and the ”≈” sign indicates no significant change. Interesting differences caused by salicylate and noise exposure in SFR are found for all auditory brain areas with the exception of the inferior colliculus (IC), where both agents are causing increased SFR. We have to be cautious here because there may be a difference between the lemniscal part of the IC, the central nucleus, and the extra-lemniscal parts such as the external nucleus and early studies were not clear about recording sites. Except for auditory nerve fibers (ANF) and secondary auditory cortex (AII) where SFRs are increased after salicylate, the tendency is for salicylate to reduce SFR in other structures (Table 1, column 1) whereas this is the opposite after noise exposure (Table 1,column 2). For noise exposure, a decrease in SFR in ANF is found and an increase nearly everywhere else. The reduction in central SFR after salicylate suggests that increases in central gain which are observed following salicylate (see next section) are not caused by homeostatic synapse mechanisms which work on SFR as well (Schaette and Kempter 2006) but likely by a specific reduction in tonic, extrasynaptic, inhibition (Richardson et al. 2011). The opposite effects of salicylate and noise exposure are also reflected in the 2-deoxyglucose (2-DG) measurements in the dorsal cochlear nucleus (DCN), but no metabolic measurement data are available for noise exposure effects in more downstream nuclei. The findings for changes in neurotransmitters are generally opposite for glutamate compared to Glycine/GABA, but the data are limited.

**Table 1:** Comparison of changes after chronic salicylate and noise exposure

Structure	SFR salicylate	**SFR NIHL**	2-DG salicylate	**2-DG NIHL**	Glutamate salicylate	**Glutamate NIHL**	Gly/GABA salicylate	**Gly/GABA NIHL**
ANF	≈^d^ ⇑^e^	**⇓** ^**x**^						
VCN		**⇑** ^**v**^						
DCN	⇓ (FF)^a^ ≈ (CW)^a^	**⇓** ^**r**^ ** ⇑** ^**s**^	⇓^n^	**⇑** ^**t**^	⇑^p^	**⇑** ^**z**^		**⇓** ^**a1**^
ICC	⇓^j^⇑^k^	**⇑** ^**a3**^	⇓(2-DG) ^m^⇑ (FDG) ^j^			**⇑** ^**y**^	⇑^p^	**⇓ → ⇑** ^**a1**^
ICX	⇑^l^		⇑^j^			**⇑** ^**y**^		
AI	≈^b^ ⇓^c,f,h^	**⇑** ^**w**^	⇑^j,m^					**⇓** ^**a2**^
AII	⇑^g^		⇑^j,m^					
Startle	+^h^	**+** ^**u**^						
Hyperacusis	+	**+** ^**u**^						

^a^Superfusion in slice (Wei et al. 2010); ^b^cat (Ochi and Eggermont 1996); ^c^Yang et al. (2007); ^d^(≤200 mg/kg, acute; Stypulkowski 1990); ^e^(≥400 mg/kg, chronic; Evans et al. 1981); ^f^cat (Zhang et al. 2011); ^g^Eggermont and Kenmochi (1998); ^h^Yang et al. (2007), Sun et al. (2009); ^i^Paul et al. (2009), ^j^ Ma et al. (2006); ^k^Bauer et al. (2008); ^l^Manabe et al. (1997), Chen and Jastreboff (1995); ^m^Wallhausser-Franke et al. (2003); ^n^Wallhäusser-Franke (1997); ^p^Peng et al. (2003); ^q^Bauer et al. (2000); ^r^fusiform cells (in vivo; Ma and Young 2006); ^s^fusiform cells FF (slice; Finlayson and Kaltenbach 2009), cartwheel cells CW (slice; Chang et al. 2002); ^t^Middleton et al. (2011) using flavoprotein imaging; ^u^Chen et al. (2013); ^v^ Vogler et al. (2011); ^w^Noreña and Eggermont (2003; 2006); ^x^Liberman and Kiang (1978); ^y^Suneja et al. (2000); ^z^Potashner et al. (1997), Whiting et al. (2009); ^a1^Suneja et al. (1998a, b), Wang et al. (2009); ^a2^Llano et al. (2012), Yang et al. (2011). ^a3^Mulders and Robertson (2009, 2013)

Vogler et al. (2011) investigated SFRs in the ventral cochlear nucleus (VCN) in the guinea pig using the same noise exposure levels known to increase SFR in the IC (Mulders and Robertson 2009). Two weeks post-trauma, the mean SFR of VCN neurons was significantly elevated compared to sham controls. This hyperactivity was more evident in primary-like and onset categories of neurons, which project to the superior olivary complex, and are involved in sound localization. Vogler et al. (2014) then recorded single-neuron SFRs in the IC of animals 2 weeks after acoustic trauma (10-kHz tone at 124 dB for 2 h) and in sham surgery controls. Following trauma, they found increased SFRs in all neuron types in the IC in regions with CFs in the peripheral hearing loss range (12–20 kHz). Thus, hyperactivity in the IC is not confined to a particular response type in contrast to findings in the cochlear nucleus.

Brozoski et al. (2007) showed that rats with behavioral evidence of tinnitus had significantly elevated neural activity in the paraflocculus of the cerebellum (PFL), as indicated by functional imaging. It was further shown that PFL activity was not elevated in normal rats listening to a tinnitus-like sound. This suggested that plastic changes in the PFL may underpin chronic tinnitus, i.e., it may serve as a tinnitus generator. Using a rat model of acoustic-trauma-induced tinnitus, Bauer et al. (2013) further examined the role of the cerebellum. It was found that PFL ablation eliminated established tinnitus without altering auditory discrimination. Similar to the ablation results, PFL inactivation with lidocaine reversibly eliminated existing tinnitus. In contrast, however, PFL ablation before tinnitus induction attenuated, but did not eliminate, tinnitus.

Knipper et al. (2010) emphasized the communality between the effects of salicylate administration and noise trauma at the cochlear level:

“While one may argue that salicylate-induced tinnitus is rather unrelated to the more common noise-induced tinnitus on the level of the hair cell, there is strong evidence from both salicylate and acoustic trauma that their deteriorative activities converge on the level of altered auditory nerve activity (…), likely upon N-methyl-D-aspartate (NMDA) receptor activation. A significant body of pharmacological evidence indicates a role of glutamate-sensitive NMDA receptor activation during stimuli-dependent excitatory events in the cochlea”.

The evidence for an involvement of NMDA receptors in tinnitus is as strong for salicylate as for noise-induced tinnitus. A dose-dependent effect of salicylate on NMDA receptor currents in neonatal spiral ganglion neurons has been described (Peng et al. 2003). Salicylate was suggested to specifically alter NMDA receptor kinetics through cyclooxygenase-induced enhancement of arachidonic acid and subsequent alteration of membrane fluidity in postsynaptic afferent synapses. Ruel et al. (2008) showed that after acute perilymphatic perfusion of salicylate, thresholds for the compound action potential (CAP) and stimulus-driven ANF activity increase together with the abolition of distortion product otoacoustic emissions (DPOAEs). These parameters did not recover in the presence of MK-801, an irreversible NMDA receptor blocker, a finding that confirms previous assumptions (Stypulkowski 1990; Müller et al. 2003) and favors the idea that salicylate-induced alterations at the level of the OHCs are not influenced by NMDA receptors. However, in the same preparation, an increase of spontaneous discharge rate of ANFs was observed after salicylate, an effect blocked by the NMDA antagonist MK-801. MK-801 alone had no effect on the spontaneous discharge rate in control animals. This may support the notion of a crucial role of NMDA receptors for trauma-induced alteration of the SFR of ANFs, although how salicylate and noise trauma yield opposite changes in the SFR of ANFs remains unclear.

In summary, the changes in SFR induced by salicylate and noise exposure differ at the level of ANFs, the DCN, and AI, but may be most similar in the IC. Since both procedures intended to induce tinnitus also increase the amplitude of startle responses giving behavioral evidence of hyperacusis (Chen et al. 2013, 2014; Sun et al. 2009), changes in the IC (and possibly AII; see Table 1) may be most closely related to hyperacusis as well as tinnitus. However, one cannot rule out the possibility that tinnitus may depend on other neural changes that may be common to salicylate and noise exposure, but are not as yet widely studied.

### Behavioral testing of animals

Research in animal models has looked for signatures of neural activity that potentially could underlie tinnitus. But how do we know that an animal has tinnitus? Typically, in behavioral test protocols, an animal is trained to respond differently to silence than to a presented sound with properties preferably similar to the expected tinnitus. Then, the animal receives a tinnitus-inducing drug such as salicylate or is exposed to noise. Some time later, the animal is assessed on its behavioral responses to continuous silence and external sound, the dominant idea being that tinnitus abolishes the notion of silence, i.e., the absence of an external sound. A state-of-the-art review is Heffner and Heffner (2012). We only list here some representative behavioral tests that have been used.

The classical behavioral techniques are based on conditioned response suppression (Estes and Skinner 1941). Jastreboff et al. (1988a, b) introduced these tests into tinnitus research. They deprived rats of water and had them continuously engaged in licking behavior during each experimental session. A constant 24-h background noise functioned as a safe-to-drink signal. The conditioned stimulus consisted of a temporary interruption of the background noise, which was paired with a mild foot shock during the training. The occurrence of silence thus slowly produced a decreased number of licks. Using this procedure, Jastreboff et al. showed that rats given salicylate after the training were less likely than control animals to stop drinking when the noise was turned off. The interpretation is that the treated animals still hear a sound when no external sound is present, i.e., they have tinnitus. Variations on shock avoidance conditioning procedures included rats learning to climb a pole during the presentation of a sound to avoid a foot shock. Animals could remain on the cage floor during quiet intervals when the shocks were turned off (Guitton et al. 2003). Following salicylate treatment, rats climbed the pole (false positive) during quiet, which was interpreted as evidence of tinnitus. Rüttiger et al. (2003) introduced a positive reinforcement technique in which responses made in the presence of sound were reinforced with a fluid reward, but not during quiet. Salicylates induced a high false response rate in quiet; the false alarm rate was equivalent to the access rate evoked by a 30-dB SPL broadband noise. In another approach, schedule-induced polydipsia (licking of a water spout induced by periodic food delivery to satiated rats) was combined with an avoidance conditioning procedure in which licking was suppressed during a sound that signaled shock (Lobarinas et al. 2004). High doses of salicylate suppressed licks-in-quiet; this was interpreted as evidence of tinnitus.

Turner et al. (2006) introduced a completely different and potentially powerful method for tinnitus screening in rats using a modified pre-pulse inhibition of the acoustic startle reflex (ASR). This method does not require training. The presence of a gap in a continuous acoustic background functioned as the pre-pulse and induced an inhibition or reduction of a loud noise-burst-induced startle reflex. The authors hypothesized that, if the background acoustic signal was qualitatively similar to the rat’s tinnitus, poorer detection of a silent gap in this background would be expected and the startle reflex would not be inhibited.

In 2011, at an international tinnitus conference in Buffalo (NY), one of us reviewed the predictions of these tests in comparison to the findings of changes in SFR and stimulus-induced firing rates and LFP amplitude in primary auditory cortex, and summarized them in the paper resulting therefrom (Eggermont (2013). It stated:

In conclusion, there are problems with the interpretation of behavioral tests in terms of tinnitus or hyperacusis if increased cortical SFR is important. There are also problems of linking increased SFR, and potentially also increased neural synchrony, in primary auditory cortex with tinnitus if the behavioral tests are reliable. Homeostatic mechanisms, affecting GABAergic activity, need to be refined to explain increased stimulus-induced activity (reflected in LFPs) in the presence of decreased SFR. Most of these problems disappear if behavioral tests only reflect increased SFR in subcortical structures. However, that still questions the role of primary auditory cortex in tinnitus perception.

Since that conference, improvements in the startle response techniques have resulted and we review here some of the new findings. Chen et al. (2013) examined the chronic effects of intense sound exposure on the acoustic startle response and its suppression by background noise containing brief gaps. They compared startle amplitudes in tone-exposed (10 kHz, 115 dB SPL, 4 h) and age-matched controls at 2**–**28 weeks post-exposure. The exposure resulted in audiometric threshold increases to 55 dB SPL at 4 and 16 kHz, and 75–80 dB SPL at 8 and 12 kHz. While both groups showed similar startle thresholds, exposed animals showed a hyperacusis-like augmentation of ASR at high stimulus levels. When the background noise contained a gap preceding the startle stimulus, ASR was suppressed in control animals, but exposed animals showed a marked weakening of gap-induced suppression of ASR. This weakening of gap-induced startle suppression is consistent with the interpretation that the gap may have been masked by tinnitus (or the startle sound was already suppressed by the continuous noise). The associated hyper-responsiveness to startle stimuli presented alone and the sensitization to background noise suggested that noise exposure leads to increases in the gain of auditory responsiveness.

Corroborating findings of the effects of hearing loss on the ASR were presented by Yurosko et al. (2014), who measured ASR amplitudes evoked by stimulus levels ranging from 57 to 120 dB SPL each day over a period of 2–3 weeks after noise exposure. At the end of this period, ABR thresholds were measured by tone pips varying from 4 to 16 kHz. All sound-exposed animals showed decreased ASR amplitudes within the first 2–3 days post-exposure. Interestingly, beginning on the third or 4th day post-exposure, the direction of change in the ASR amplitudes reversed in 3 of the 4 exposed animal groups. Startle amplitude increased above control levels, and the degree of the increase was in proportion to the degree of maximal threshold shift, which varied across groups between 36 and 62 dB. However, animals with threshold shifts exceeding 75 dB showed no ASR reversal. The results suggest that noise exposure induces hyperacusis-like enhancements of startle with moderate threshold shift (up to 62 dB), but severe threshold shift (75 dB or more) decreases startle amplitude.

Temporary threshold shift (TTS)-causing noise exposure can result in immediate, permanent partial degeneration of the auditory nerve in mice despite complete threshold recovery and lack of hair cell damage (Kujawa and Liberman 2009; Lin et al. 2011); Hickox and Liberman (2014) measured the ASR and prepulse inhibition (PPI) thereof in mice exposed for 2 h either to a “neuropathic” (100 dB SPL) noise or to a “nonneuropathic” (94 dB SPL) noise and in unexposed control mice. Mice with loss of ribbon synapses that innervate high-threshold ANFs, resulting in loss of activity in these fibers (cochlear neuropathy), displayed hyper-responsivity to sound, evidenced by enhanced ASR and PPI, while exposed mice without neuronal loss showed control-like responses. Gap-PPI tests revealed limited gap detection deficits in mice with cochlear neuropathy, which is inconsistent with the presence of tinnitus “filling in the gap.” Considering the rapid post-exposure onset of both cochlear neuropathy and exaggerated startle-based behavior, the results suggest a role for cochlear primary neuronal degeneration in the generation of hyperacusis.

These contrasting interpretations beg for a validation of the ASR in humans with tinnitus and with or without hyperacusis, and indeed a recent study indicates that problems with the ASR exist. Fournier and Hébert (2013) investigated the gap startle paradigm in human participants with high-frequency tinnitus. They tested 15 adults with bilateral high-frequency tinnitus but normal hearing at standard audiometric frequencies and 17 matched controls without tinnitus. The psychoacoustic characteristics of the tinnitus spectrum (pitch and loudness) were assessed. The startle task consisted of startle alone, PPI, and a gap-in-noise condition using a low-frequency band-pass background noise (control condition, CF = 500 Hz) and high-frequency band-pass noise (tinnitus condition, CF = 4 kHz). All measurements were repeated after several months. At both measurements, participants with tinnitus displayed normal PPI but higher reactivity to the startle sounds compared to controls. In addition, the tinnitus group displayed a deficit in gap processing during the high (tinnitus) background noise and also during the low control background sound; the expected difference between these conditions did not reach significance. The lack of frequency specificity in gap suppression did not support the hypothesis that tinnitus “fills in the gap”, which has been proposed from animal data. The authors suggested instead that the higher reactivity to startle reflected hyperacusis, while the frequency-nonspecific deficit in gap processing reflected abnormal cortical auditory temporal processing in individuals with tinnitus.

###  Gap detection in animals and humans

**Fig 2:**
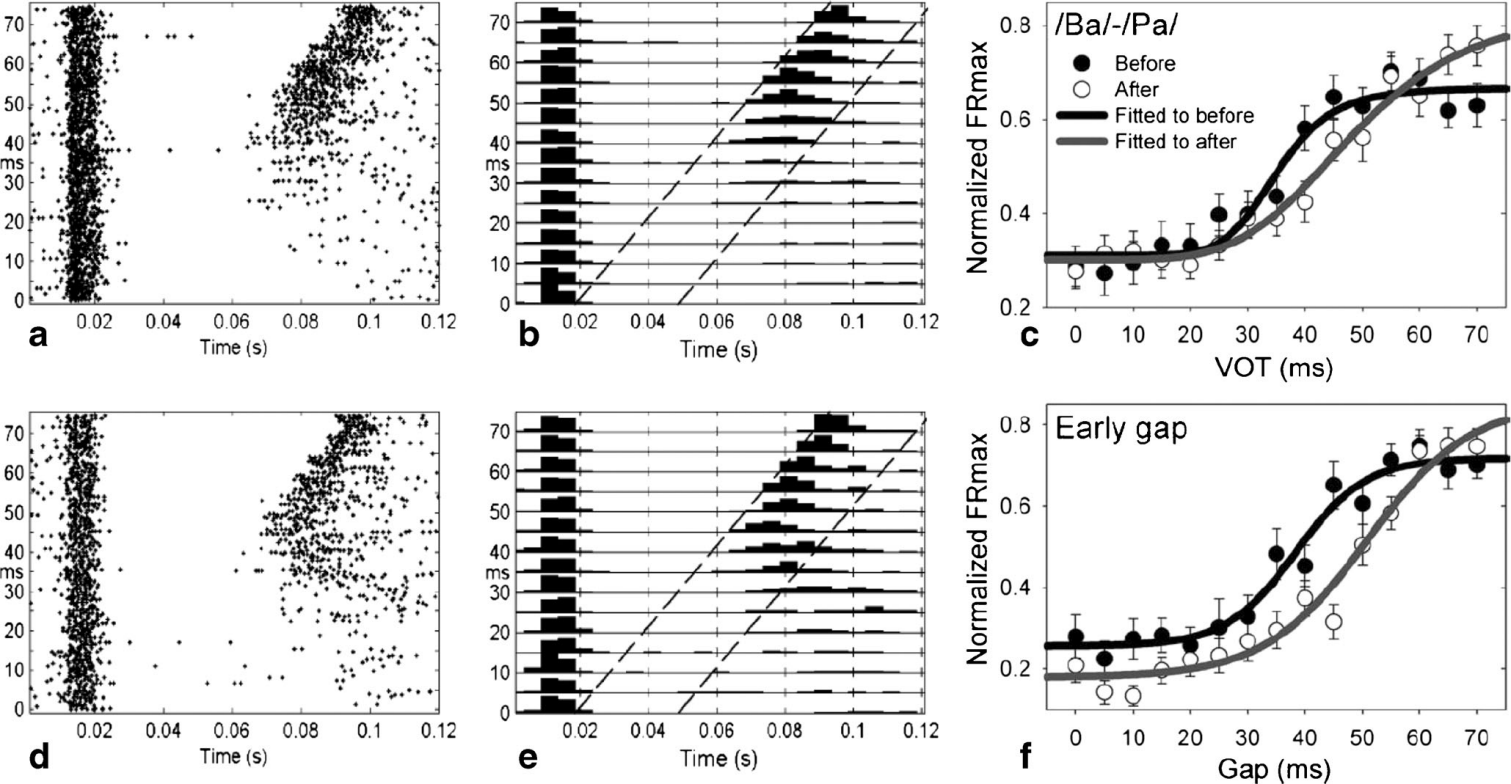
A comparison of the responses to a /ba/–/pa/ continuum (**a**–**c**) and early gap (**d**–**f**) conditions from the same recording site. Dot displays (*left column*) and PSTH (*middle column*) are organized vertically according to VOT or gap duration and horizontally for time since the onset of the leading noise burst. Time windows for evaluation of the PSTHs to the trailing stimulus are selected (between *dot lines*) according to VOT or gap duration and the latency of peak response for the leading noise burst. Compare in the *right panels* the average normalized maximum firing rate for the vowel (*top*) and trailing noise burst after the early gap (*bottom*) obtained before (*filled circles*) and after (*open circles*) the acoustic trauma (±SE). The *sigmoid curves* provide the best statistical fit to the data. Note that fitted curves for both the /ba/–/pa/ continuum and the early gap condition are shifted toward longer VOT or gap duration. *FRmax* maximum firing rate. From Tomita et al. (2004)

The abnormal auditory temporal processing hypothesis suggests that it may be important to consider the effects of hearing loss alone on gap detection thresholds, since impaired startle suppression could arise from reduced sensitivity to gaps (impaired temporal processing), as well as from filling-in effects from tinnitus. We (Tomita et al. 2004) showed that noise-induced hearing loss causes a decrease in neural temporal resolution. We investigated the effect of an acute NIHL (5 or 6 kHz at 115–120 dB SPL for 1 h) on the representation of a voice onset time (VOT) and gap-duration continuum in primary auditory cortex of the ketamine-anesthetized cat. Multiple single-unit activity related to the presentation of a /ba/–/pa/ continuum—in which VOT was varied in 5-ms steps from 0 to 70 ms—was recorded from the same sites before and after an acoustic trauma using two 8-electrode arrays. We also obtained data for gaps, of duration equal to the various VOT values, embedded in noise 5 ms after the onset. The changes in the maximum firing rate for /ba/–/pa/ continuum as a function of VOT matched the psychometric function for categorical perception of /ba/–/pa/ modeled by a sigmoid function. An acoustic trauma made the sigmoid fitting functions shallower, and shifted them toward higher values of VOT (Fig. 2). The less steep fitting function may be a neural correlate of an impaired psychoacoustic temporal resolution, because the ambiguity between /ba/ and /pa/ should consequently be increased.

Behavioral detection of gaps embedded in continuous broadband noise presented at 20, 30, 40, or 60 dB SPL was recently tested in rats by Radziwon et al. (2014). The rats were tested using a go/no-go operant conditioning procedure to detect a silent gap embedded within the broadband noise as well as to determine broadband noise thresholds in quiet. The rats were tested once per week with either a single injection of sodium salicylate (200 mg/kg), previously shown to induce tinnitus in rats, or an equivalent volume of saline. Mean gap-detection thresholds in saline increased from approximately 2 ms for stimuli presented at 60 dB to approximately 5 ms at 20 dB. Note that the previous paragraph showed minimum gap durations of 30–40 ms. In the Tomita et al. (2004) study, the gap was placed 5 ms after noise onset; however, when the gap is inserted about 200 ms after the noise onset, the minimum gaps are also in the 2–5 ms range (Eggermont 1999). Salicylate had little or no effect on gap thresholds at higher noise levels (30–60 dB SPL); the rats (presumed to have tinnitus) could always detect gaps longer than 10 ms at these sound levels. Since salicylate produced a mean hearing loss of 17 dB SPL in the broadband noise detection experiment, the effects of salicylate seen in the gap detection experiment can be explained by hearing loss, which impairs temporal resolution at low sensation levels. These results suggested that gap detection deficits from salicylate are the result of hearing loss alone and not caused by tinnitus filling in the silent gaps.

Gap detection in hearing-impaired human listeners is degraded relative to that of normal-hearing listeners when compared at equal sound pressure level (Reed et al. 2009). Campolo et al. (2013) asked hearing-impaired subjects with tinnitus if they could perceive 50-ms silent intervals presented in a narrow-band noise, which was located in frequency range above, below or at the subject’s tinnitus pitch. The same tests were performed on normal hearing subjects without tinnitus. All subjects, with and without tinnitus, could detect the 50-ms gaps. Thus, using the stimulus parameters similar to those employed in animal and human gap-startle studies, they found that the tinnitus percept does not fill in the silent interval in a perceptual gap detection task. Similar results were obtained by Gilani et al. (2013) who tested 20 tinnitus patients and 20 healthy volunteers without tinnitus, all with normal auditory thresholds (≤20 dB nHL) with gaps in noise. There were statistically significant increases in an approximate threshold value of gap detection in the patients group, both in right and left sides. These results identified auditory temporal resolution difficulties in patients with tinnitus, meaning that, in spite of normal auditory thresholds, there may be some possibility of abnormality in central auditory temporal processing.

The studies reviewed in this section suggest that impaired temporal processing in subjects with tinnitus (whether human or animal) is a factor that may contribute to reduced startle suppression by temporal gaps in background sound. Sensitization of startle responses after noise exposure or salicylate is a further factor that could modulate the extent of startle suppression induced by gaps after these procedures. However, it should be noted that several animal studies did find significant frequency specificity in the extent to which gaps in background sound suppress the startle response, with peak suppression failure coinciding with frequencies where tinnitus was expected to have occurred (Dehmel et al. 2012; Koehler and Shore 2013; Turner et al. 2006). This is a putative hallmark of the presence of tinnitus. Results obtained by using gap startle suppression to identify animals experiencing tinnitus have been informative about neural pathways and mechanisms that may be involved in tinnitus, but need to be evaluated in the light of other interpretations.

### Acute versus chronic hyperactivity; timelines for centralization

Longenecker and Galazyuk (2011) induced tinnitus in a group of mice by exposure to a 116-dB SPL one-octave band noise centered at 16 kHz during 1 h under anesthesia. The tinnitus was assessed behaviorally by measuring gap-induced suppression of the acoustic startle reflex. They found that a vast majority of the sound-exposed mice (86 %) developed behavioral signs of tinnitus. This was a complex, long-lasting, and dynamic process. On the day following exposure, all mice demonstrated signs of acute tinnitus over the entire range of sound frequencies used for testing (10**–**31 kHz). However, 2**–**3 months later, behavioral evidence of tinnitus was evident only at a narrower frequency range (20**–**31 kHz) representing a presumed chronic condition. Extracellular recordings confirmed a significantly higher SFR in IC neurons in sound-exposed compared to control mice. Sun et al. (2012) studied the effects of noise exposure (narrow band noise, 12 kHz, 120 dB SPL, 1 h) on the neural responses of the IC and the auditory cortex (AC), and the behavioral sound reaction in awake rats. Noise exposure induced a decrease of sound evoked LFP in the IC. This is likely a transient phenomenon since at 12 h post-trauma, increased SFRs can be found in IC (Mulders and Robertson 2013). In contrast, significant increases in sound evoked LFPs and firing rates were found in AC immediately after the noise exposure. This suggested that the acute noise-exposure-induced hyperexcitability of AC was likely not driven by subcortical activity. The behavioral consequence of this finding was compared to the amplitude of the acoustic startle response before and after noise exposure in a separate group of rats. Although noise exposure caused a hearing loss of about 70 dB for frequencies of 16 and 24 kHz and ~40 dB for 4 and 8 kHz, the acoustic startle amplitude at the super-threshold level (120 dB SPL) was significantly increased. These results suggest that noise exposure can cause exaggerated sound reactions which may be related with the enhanced responsiveness of the AC neurons. This phenomenon may be related with noise-induced hyperacusis. One has to note that the acoustic startle reflex results from a subcortical circuit and one would thus expect otherwise because of the reduced LFP amplitude in IC. Modulation of the reflex via secondary auditory cortex, however, is possible (Eggermont 2013).

The time of onset of increased SFRs was investigated (Mulders and Robertson 2013) in the IC of guinea pigs subjected to unilateral acoustic trauma (exposure to a 10-kHz tone at 124 dB for 2 h). Hyperactivity was present by 12 h post-acoustic trauma, whereas data obtained within approximately 4 h of the cessation of acoustic trauma found no evidence of increased SFRs. Thus, increased SFRs in the IC occur at some time between 4 and 12 h post-trauma and this is a relatively rapid plastic event beginning within hours rather than days post-cochlear trauma (as found previously in the DCN by Kaltenbach et al. 2000). The delayed onset is comparable with findings in the cat primary auditory cortex (Noreña and Eggermont 2003), albeit that they found increased SFR already after 2 h post-trauma. SFRs in IC did not show any further systematic increase between 12 h and up to 2 weeks post-acoustic trauma, again in agreement with the cortical findings by Noreña and Eggermont (2003, 2006). At recovery times of 12 and 24 h, hyperactivity was widespread across most regions of the IC, but at longer recovery times, it became progressively more restricted to CF regions corresponding to those of the cochlea with persistent damage.

Abnormally elevated spontaneous neural activity has been found in the DCN of animals with behavioral evidence of tinnitus. However, DCN ablation after 3–5 months post-trauma failed to reduce established tinnitus (Brozoski and Bauer 2005; see also the findings of Mulders and Robertson 2009, 2011 described below). Thus, the DCN may serve as a necessary trigger zone rather than a chronic generator of tinnitus. To test this hypothesis, Brozoski et al. (2012) used lesion procedures identical to those that failed to decrease pre-existing tinnitus but now prior to tinnitus induction. Young adult rats were trained and tested using a behavioral procedure shown to detect tinnitus. Tinnitus was induced by a single unilateral high-level noise exposure. Bilateral dorsal DCN lesions made before high-level noise exposure prevented the development of tinnitus, consistent with a role for the DCN in the generation of tinnitus but not its maintenance.

Manzoor et al. (2012) investigated the extent to which IC hyperactivity is dependent on input from the contralateral DCN by comparing SFR in the IC of noise-exposed and control hamsters before and after ablation of the contralateral DCN. One group of animals was binaurally exposed to intense sound (10 kHz, 115 dB SPL, 4 h), whereas the control group was not. Both groups were studied 2–3 weeks later by mapping SFRs along the tonotopic axis of the IC to confirm induction of hyperactivity. Spontaneous activity was then recorded at a hyperactive IC locus over two 30-min periods, one with DCNs intact and the other after ablation of the contralateral DCN. Ablation of the DCN resulted in major reductions of IC hyperactivity, confirming the need for SFR input to the IC as also found by Mulders and Robertson (2009). Levels of post-ablation activity in exposed animals were similar to the levels of activity in the IC of control animals, indicating an almost complete loss of hyperactivity in exposed animals. The results suggest that hyperactivity in the IC is dependent on support from extrinsic sources that include and may even begin with the DCN. Interestingly, as we have seen, the increased SFR in the VCN (Vogler et al. 2011) observed in primary-like and onset type of neurons appears not to be sufficient to provide for increased SFR in IC (provided that the VCN was not affected by the ablation procedure in the cited studies), further implicating neural drive from the DCN in the establishment of IC hyperactivity. Mulders and Robertson (2011) further showed that hyperactivity in the IC induced by noise trauma was abolished by resection of the cochlea up to 6 weeks after noise trauma but not if resection was performed later. These findings demonstrate that the increased SFR that develops after acoustic trauma transitions from an early stage, when it is dependent on continued peripheral afferent input, to a later stage in which the hyperactivity is intrinsically generated within the central nervous system.

This evidence for centralization of the neural changes underlying tinnitus over the first few months after the trauma suggests further that increased central gain resulting from noise exposure has to work upon both driven and spontaneous input arising either from the damaged cochlea (Mulders and Robertson 2009) or the DCN (Manzoor et al. 2012). This is a signature of homeostatic forms of plasticity proposed to underlie increased SFR in DCN and other neural changes related to tinnitus in central auditory structures (Schaette and Kempter 2006).

### Neural changes in nonauditory pathways

Functional imaging in humans (reviewed in “Human studies”) has been the main source of information about changes that occur in nonauditory structures in individuals experiencing tinnitus. However, animal investigations have also revealed changes taking place beyond central auditory pathways. Early immune-labeling studies by Wallhausser-Franke et al. (2003) and Mahlke and Wallhäusser-Franke (2004) showed that high doses of sodium salicylate, known to induce tinnitus, not only activate central auditory structures but also the amygdala, a non-auditory limbic structure associated with emotion that can modulate neuron sensitivity and plasticity in the auditory cortex via projections through the basal forebrain. To identify the electrophysiological changes occurring in several non-auditory regions, Chen et al. (2014) treated rats with 250 mg/kg salicylate and recorded the local field potentials and multiunit firings from the striatum (Str), lateral amygdala (LA), hippocampus (HC), and cingulate cortex (Cg). Salicylate treatment (200 mg/kg) produced behavioral evidence of tinnitus (measured by a two-alternative forced choice procedure) and hyperacusis-like behavior, as reflected enhanced startle responses to noise bursts. Salicylate (250 mg/kg) enhanced sound-evoked neural activity in the Str, LA, and HC, but not the Cg. Although salicylate significantly enhanced sound-evoked responses, the mean SFR in these regions was not significantly increased or was even decreased in some cases. Interestingly, the enhancement of sound-evoked electrophysiological activity occurred predominantly at the mid-frequencies, likely reflecting an up-shift of the characteristic frequency of low-frequency neurons and down-shift of high-CF towards the middle frequencies in auditory cortex (Chen et al. 2012; Stolzberg et al. 2011). The tonotopically over-represented region could potentially lead to a mid-frequency pitch tinnitus. The enhanced neural activity in the LA and HC, regions involved in emotion and memory, may contribute to the negative effect that patients associate with their tinnitus.

In a subsequent study, Salvi and Chen (2014) compared these changes in nonauditory regions after salicylate-induced tinnitus with those observed after intense high-frequency noise exposure. The intense noise exposure enhanced the responses of neurons in the amygdala to frequencies below the noise band, while the responses to frequencies within and above the noise damaged area were attenuated or abolished. Interestingly, in a few animals, the enhancement of sound evoked activity was observed at frequencies near the edge of the hearing loss reflecting plastic reorganization. In contrast to salicylate, the noise-induced enhancement of the auditory response was not observed in the striatum. Following high-frequency intense noise exposure, the amygdala undergoes physiological changes possibly related to noise-induced tinnitus and hyperacusis. However, noise exposure does not affect the striatum in the same way as salicylate, suggesting that the striatum may not be involved in noise-induced tinnitus and/or hyperacusis.

Changes in nonauditory brain regions in the presence of tinnitus could reflect outputs from central auditory pathways or be mediated indirectly by inputs from auditory pathways to neuromodulatory systems in the basal forebrain (nucleus basalis of Meynert) and midbrain (locus coeruleus, reticular formation, raphe nuclei) that are important in attention, emotion, and plasticity (Roberts et al. 2013). A final common pathway of tinnitus consisting of regions that are activated by all cases of tinnitus had been proposed much earlier (Shulman 1995) in which the amygdala and hippocampus have a fundamental function together with the parabrachial nucleus and insula.

### Tinnitus and neural plasticity

Feldman (2009) described 5 common components of sensory plasticity in neocortex. These are (1) rapid response depression to deprived inputs, (2) slow response potentiation to spared inputs flanking the deprived ones, (3) rapid potentiation of responses to active inputs during normal use, (4) rapid potentiation of responses paired with reinforcement, and (5) slow homeostatic regulation of cortical activity in response to substantial increase or decrease in sensory input (for application to tinnitus see Yang et al. 2011). Typically, experience-dependent plasticity is composed of two opposing but complementary forces: one that modifies neuronal circuits progressively by creating selective differences between individual elements and another that regulates circuit properties to stabilize the overall activity of the network (Turrigiano 1999). The first force is Hebbian-like and consists of correlation-based mechanisms that progressively and rapidly modify network properties. The second is produced by slower homeostatic mechanisms that promote network stability. Several activity-dependent molecular signals have been proposed to have a role in synaptic scaling, including brain-derived neurotrophic factor (BDNF), cytokine tumor-necrosis factor α (TNFα), and the effector immediate-early gene product Arc (Turrigiano 2007; Knipper et al. 2010).

A potential correlation-based mechanism is spike time-dependent plasticity (STDP) that can take several forms. The most common are termed Hebbian and anti-Hebbian (Feldman 2012). Hebbian STDP is induced when synaptic activity preceeds a post-synaptic spike potentiates the synapse while synaptic activity following a postsynaptic spike depresses the synapse. In contrast, anti-Hebbian STDP is induced when synaptic activity precedes a post-synaptic spike and depresses the synapse while synaptic activity following a postsynaptic spike potentiates the synapse. Dehmel et al. (2012) demonstrated in vivo that DCN bimodal plasticity is stimulus timing-dependent with Hebbian and anti-Hebbian timing rules that reflect the in vitro spike timing-dependent plasticity (Tzounopoulos et al. 2004). In a subsequent in vivo study, Koehler and Shore (2013) assessed this bimodal STDP in a tinnitus model. Guinea pigs were exposed to a narrowband noise that produced a temporary elevation of ABR thresholds. A total of 60 % of the guinea pigs developed tinnitus as indicated by gap-induced PPI of the acoustic startle response. After noise exposure and tinnitus induction, STDP was measured by comparing responses to sound before and after paired somatosensory and auditory stimulation presented with varying intervals and orders. In comparison with sham and noise-exposed animals that did not develop tinnitus, timing rules in verified tinnitus animals were more likely to be anti-Hebbian with a broader window for those bimodal intervals in which the neural activity showed enhancement. Furthermore, units from exposed animals with tinnitus were less suppressed than either sham animals or exposed animals without tinnitus. The broadened timing rules in the enhancement phase in animals with tinnitus, and in the suppressive phase in exposed animals without tinnitus, was in contrast to narrow, Hebbian-like timing rules in sham animals. Because DCN neurons are more responsive to somatosensory stimulation following hearing damage (Shore et al. 2008), bimodal plasticity in DCN may play a role in somatic tinnitus, the modulation of tinnitus pitch and loudness by pressure or manipulation of the head and neck. The observed effect that spinal trigeminal nucleus stimulation preceding tone stimulation shifts from suppression in normal animals to enhancement in animals with PPI-ASR evidence of tinnitus (Dehmel et al. 2012) suggests that bimodal plasticity may contribute to DCN hyperactivity in tinnitus. A subsequent study using the same methods (Basura et al. 2014) reported STDP in the auditory cortex with a similar shift toward predominantly anti-Hebbian timing rules after 15 min of bimodal pairing in animals expressing behavioral evidence of tinnitus.

Singer et al. (2013) examined both the impact of different degrees of cochlear damage and the influence of stress priming on tinnitus induction. They used a behavioral animal model for tinnitus designed to minimize stress, assessed the ribbon synapses in inner hair cells (IHCs) as a measure for deafferentation, and used auditory brainstem responses (ABRs) to detect differences in stimulus-evoked neuronal activity. In addition, they measured the expression of the activity-regulated cytoskeletal protein, Arc (a marker for activation of plasticity in the brain), to identify long-lasting changes in network activity within the basolateral amygdala (BLA), hippocampal CA1, and auditory cortex. They observed that IHC ribbon loss led to behavioral signs of tinnitus when (1) ABR amplitude-level functions remained sub-normal for waves 4 and 5, reflecting an absence of synaptic gain increase, and (2) when Arc was not mobilized in the hippocampal CA1 and AC. Reduction of Arc results in loss of the normal scaling responses to changes of neuronal activity. If, however, ABR wave amplitudes were functionally normal despite the hearing loss, suggesting increased synaptic gain, and Arc was mobilized, tinnitus did not occur (as reflected by absent gap-PPI-ASR in Hickox and Liberman 2014). The same group (Rüttiger et al. 2013) found that, although both tinnitus and non-tinnitus animals exhibited a reduced ABR wave I amplitude (generated by ANFs), IHC ribbon loss and high-frequency hearing impairment were more severe in tinnitus animals. This was associated with significantly reduced ABR wave 4 and 5 amplitude and less intense staining of *Arc* mRNA and protein in the AC. This again suggests that tinnitus may be linked to a failure to adapt central circuits to reduced cochlear input.

Largely corroborating these findings, Hu et al. (2014) used the gap PPI of the acoustic startle response to test for salicylate-induced tinnitus-like behavior in rats. Rats received 200 mg/kg of salicylate daily for up to 14 days. Expression of the *Arc* gene and the early growth response gene-1 (*Egr-1*) gene were decreased in the IC and AC. Expression of NMDA receptor subunit 2B was increased and all these changes returned to normal 14 days after treatment with salicylate ceased. This long-time administration of salicylate induced tinnitus markedly but reversibly caused neural plasticity changes in the IC and the AC. Decreased expression of *Arc* and *Egr-1* might be involved with instability of synaptic plasticity in tinnitus. The failure to mobilize Arc in the cortex suggested that tinnitus is linked to a failure to adapt central circuits to reduced cochlear input. One has to keep in mind that application of salicylate damages spiral ganglion cells in culture (Deng et al. 2013), so secondary degeneration of ANFs in vivo cannot be ruled out.

The observations of these four studies are provocative because they do not align in any straightforward way with the view that tinnitus and hyperacusis reflect an up-scaling of central neural responses consequent on homeostatic plasticity operating in deafferented auditory pathways. They are consistent, however, in associating tinnitus with ribbon loss in IHCs, and with changes in protein synthesis related to neural plasticity. One hypothesis could be that mobilization of Arc and other proteins when it occurs may protect from tinnitus after damage to ribbon synapses, while up-scaling of neural responses by homeostatic plasticity may be an independent process more closely linked with hyperacusis. This suggests, in contrast to the findings of Vogler et al. (2011) and Mulders and Robertson (2009, 2011), that SFR increases resulting from central gain increases are not causal to behavioral evidence of tinnitus. Either increased SFR is not sufficient for tinnitus to occur, or the interpretation of the behavior model results is inconsistent with the role proposed for increased SFR, i.e., tinnitus filling the gap.

The results of Kraus et al. (2011) align with the hypothesis just mentioned, namely that tinnitus is related to a comparatively weaker induction of plasticity-related protein synthesis. These authors unilaterally exposed rats to narrow-band noise centered at 12 kHz at 126 dB SPL for 2 h and sacrificed them 10 weeks later for evaluation of synaptic plasticity based on the expression of growth-associated protein 43 (GAP-43) in the cochlear nucleus. Noise-exposed rats along with age-matched controls were screened for tinnitus-like behavior with gap PPI of the acoustic startle before, 1–10 days after, and 8–10 weeks after the noise exposure. All nine noise-exposed rats showed similar patterns of severe hair cell loss at high- and mid-frequency regions in the exposed ear. They showed strong up-regulation of GAP-43 in auditory nerve fibers and pronounced shrinkage of the VCN on the noise-exposed side, and strong up-regulation of GAP-43 in the medial ventral VCN, but not in the lateral VCN or the DCN. GAP-43 up-regulation in VCN was significantly greater in noise-no-tinnitus (NT) rats than in noise-tinnitus (T) rats. These results suggest that noise-induced tinnitus is suppressed by strong up-regulation of GAP-43 in the medial VCN.

This evidence for differences in the expression of proteins related to neural plasticity between animals with and without behavioral signs of tinnitus raises the question of a role for trophic factors (corticosteroids and corticotrophins) in neural changes underlying tinnitus. Corticotrophin-releasing hormone (CHR) is released in response to stress from the median eminence of the hypothalamus and acts on the pituitary, but is also expressed in the amygdala and hippocampus, which are structures important in emotion and memory. CRH binds to G-protein-coupled receptors and in the hippocampus primes changes in LTP, while in the amygdala CRH release enhances memory consolidation (see Joëls and Baram 2009 for a review). Corticosteroids are secreted by the adrenal glands during activation of the hypothalamic-pituitary axis (HPA) by stress and bind to glucocorticoid receptors (GRs) and mineralocorticoid receptors (MRs), which are widely distributed in the brain. GRs and MRs are found in prefrontal cortex, limbic regions including the hippocampus, amygdala, and lateral septum, and also in the cochlea; in the latter structure, GRs are found on spiral ganglion neurons and IHCs (although fewer on OHCs). On binding the steroid hormone, MRs and GRs translocate to the cell nucleus (de Kloet et al. 2005) where they act as regulators of gene transcription, giving them a role in functional and structural neural plasticity on short and long time scales. On a short time scale, GR activation protects against threshold shifts induced by noise trauma in a mouse model (Meltser and Canlon 2011), and may play a role in modulating vulnerability to noise trauma which has been reported over the circadian cycle (Meltser et al. 2014), possibly by influencing BDNF expression (Numakawa et al. 2013). In the longer term, persistent stress reported as emotional exhaustion by tinnitus sufferers (Hébert et al. 2012), and reflected by elevated cortisol levels in tinnitus patients reporting high distress (Hébert et al. 2004), could initiate a cascade of effects leading eventually to alterations in dendrite and spine morphology (Joëls et al. 2007) and structural changes reported in functional imaging studies of tinnitus (see later).

One question not previously addressed is whether neural changes taking place in the auditory cortex in animals expressing behavioral evidence of tinnitus after noise exposure are modulated by a down-regulation of inhibition inherited from subcortical pathways. Sametsky et al. (2014) examined GABAergic inhibition in MGB of three groups of rats: control (C), sound-exposed with behavioral evidence (PPI-ASR) of tinnitus (T), and sound-exposed animals showing no behavioral evidence of tinnitus (NT). Using in vitro whole-cell recordings of thalamocortical neurons, extrasynaptic tonic GABA_A_ receptor (GABA_A_R) currents were evoked by bath application of the subunit selective agonist, gaboxadol (0.1-10 μM), in the presence of glutamatergic blockers. Two months following tinnitus-inducing sound exposure, significant (*p* = 0.02) increases in tonic GABA_A_R currents were observed in MGB neurons from tinnitus compared to control animals contralateral to the exposure. Results in NT neurons were not statistically different from those of C or T neurons. Analysis of spontaneous inhibitory postsynaptic potentials revealed no differences in measured parameters, implying that net synaptic GABA transmission (phasic inhibition) in MGB was not reduced in T animals. There was no down-regulation of inhibitory transmission in the MGB in animals expressing behavioral evidence of tinnitus. In contrast, tonic GABA_A_R currents were elevated in MGB neurons, lending some support to the notion that cortical dynamics putatively associated with tinnitus, such as increased SRF and neural synchrony in cortical neurons affected by hearing loss (Eggermont and Roberts 2004), may reflect disinhibition of the affected cortical regions (Scholl and Wehr 2008) by hyperpolarization of thalamic neurons consequent on deafferentation (Llinás et al. 2005).

## Human studies

Animal studies of tinnitus measure single- and multi-unit neural activity, and LFPs with precise temporal and anatomical resolution, but are limited by the necessity of inferring the presence or absence of tinnitus and/or hyperacusis from indirect behavioral methods. In contrast, hemodynamic and electromagnetic imaging methods, which are applicable to humans provide either poorer spatial (MEG, EEG) or temporal (PET, fMRI) resolution compared to physiological experiments in animals. However, human studies can manipulate the presence or absence of tinnitus and hyperacusis based on verbal reports from the participants and assess both with psychoacoustic methods. Functional imaging is also well suited to investigating brain network behavior associated with tinnitus and its correlated conditions including hyperacusis and emotional distress.

### Changes in auditory pathways

Numerous animal studies reviewed in the previous section have demonstrated elevated spontaneous and sound-evoked activity in brainstem auditory pathways. In humans with and without tinnitus, Gu et al. (2012) assessed auditory nerve and brainstem function in response to sound using auditory brainstem responses. Tinnitus subjects showed reduced wave I amplitude (indicating reduced auditory nerve activity) but enhanced wave V (reflecting elevated input to the inferior colliculi) compared with non-tinnitus subjects matched in age, sex, and pure-tone threshold. Compared with a third cohort of younger, non-tinnitus subjects, both tinnitus and non-tinnitus groups showed elevated thresholds above 4 kHz and reduced wave I amplitude. Animal lesion and human neuroanatomical data combined indicated that waves III and V in humans reflect activity in a pathway originating in the VCN and with spherical bushy cells (SBC) in particular. SBC output is involved in sound localization and projects to the medial superior olive (see also Vogler et al. 2011 for supporting animal data). This implies a role for the VCN in tinnitus. The reduced wave I and enhanced wave V observed in tinnitus subjects in this study revealed changes in central gain appearing early, i.e., below the inferior colliculus, in the auditory pathway. The results also align with the behavioral data described earlier (Hébert et al. 2013), revealing steeper loudness growth functions in individuals reporting tinnitus with normal audiograms (thresholds ≤15 dB HL for ≤8 kHz) compared to their matched controls.

A recent comprehensive study by Melcher and colleagues (Gu et al. 2010) examined auditory brain areas involved in chronic tinnitus and hyperacusis, which frequently but not always co-occur in patients reporting either condition. Patients with and without tinnitus, all with clinically (≤8 kHz) normal hearing thresholds, underwent both behavioral testing to assess their sound-level tolerance (i.e., the presence or absence of hyperacusis) and fMRI to measure sound-evoked activation of central auditory centers. Despite receiving identical sound stimulation levels, subjects with hyperacusis showed elevated evoked activity in the IC, MGB, and primary auditory cortex (AI) compared with subjects with normal sound tolerance. This reflects the increased gain for processing external auditory stimuli. AI, but not subcortical centers, also showed elevated activation specifically related to tinnitus, i.e., in the absence of hyperacusis. The authors hypothesized that the tinnitus-related elevations in cortical activation could reflect undue attention drawn to the auditory domain. This is consistent with the lack of pure tinnitus-related effects subcortically where activation is typically less modulated by the attentional state. Given its role in modulating the sensitivity of cortical neurons to their afferent inputs and its consequences for neural plasticity, a mechanism for auditory attention (particularly one involving the basal forebrain cholinergic system) could be expected to play a role in forging neural network activities that underlie tinnitus percepts. That tinnitus is itself a persistent audible percept could be taken as prima facie evidence for an involvement of auditory attention mechanisms (Roberts et al. 2013).

Langers et al. (2012) investigated tonotopic maps in primary auditory cortex of 20 healthy controls and 20 chronic subjective tinnitus patients. The goal was to test the hypothesis, proposed on the basis of animal and previous human studies (Eggermont and Roberts 2004), that tinnitus results, among others, from an abnormal tonotopic organization of the auditory cortex. Subjects were recruited from the hospital’s tinnitus outpatient clinic as well as from advertisements in various media. The patients reported no history of neurological or psychiatric disorders, and were not undergoing tinnitus treatment at the time of the study. All subjects were selected to have normal or near-normal hearing up to 8 kHz. The study found no evidence for a reorganization of cortical tonotopic maps. This is perhaps not surprising since there was no appreciable hearing loss ≤8 kHz. It had been previously shown that in cats there is no reorganization of the cortical tonotopic map for hearing losses ≤25 dB (Rajan 1998; Seki and Eggermont 2002). In another study of map reorganization in tinnitus, Wienbruch et al. (2006) investigated tonotopic gradients in 28 tinnitus patients and 17 controls using the auditory steady-state response (ASSR) evoked by 40-Hz AM tones of different carrier frequencies and recorded by MEG. The cortical sources of the ASSR localize to AI with a frequency organization reflecting summation across tonotopic maps sharing a common low-frequency border situated laterally in Heschl’s gyrus (Pantev et al. 1996; Gander et al. 2010a). Sound thresholds measured by attenuation in the MEG dewar were elevated by about 15–20 dB in the tinnitus group compared to controls, indicating the presence of hearing loss in this group. When normalized across coordinates from 3 days, frequency (tonotopic) gradients for the ASSR showed a significant high-frequency posterior-medial to low-frequency antero-lateral trend in control subjects that was flattened from 1,296 to 6,561 Hz (the highest frequency tested) in the tinnitus group, indicating a loss of tonotopic order in this frequency region in tinnitus subjects, particularly in the right hemisphere. Taken together, these results from different imaging methods support the conclusion that map reorganization in tinnitus may depend on the extent of hearing loss in tinnitus subjects. The results of Langers et al. (2012) further indicate that macroscopic map reorganization in auditory cortex is not a requirement for tinnitus to occur, nor are clinically abnormal audiograms.

The imaging results reported by Wienbruch et al. (2006) and Gu et al. (2012) in tinnitus subjects, and psychoacoustic data localizing tinnitus to high-frequency sounds (Noreña et al. 2002; Roberts et al. 2008; Fig. 1), suggest that there is something special about this frequency region of AI in tinnitus subjects. Consistent with this, Paul et al. (2014) found that modulation of ASSR amplitude by top–down attention was impaired in tinnitus compared to control subjects when the ASSR was evoked by a 5-kHz sound in the tinnitus frequency region (TFR) of the tinnitus group, but not when the ASSR was evoked by a 500-Hz sound below this region. In contrast, modulation of the N1 potential localizing to nonprimary auditory cortex (AII) was impaired at both frequencies in the tinnitus but not the control groups. Paul et al. suggested that tinnitus-related neural activity occurring in the 5-kHz but not the 500-Hz region of tonotopic AI disrupted attentional modulation of the 5-kHz ASSR in tinnitus subjects, while tinnitus-related activity in AI distributing to AII where tonotopic organization is weak or absent (Lütkenhöner et al. 2003) impaired modulation of N1 at both sound frequencies. These findings support the hypothesis of Gu et al. (2010) that neural activity in cortical auditory attention networks is modified by the presence of tinnitus, and indicate further that this activity may be frequency specific in primary but not in nonprimary cortical regions.

In a study related to this theme, Roberts et al. (2013) investigated whether the rules of neural plasticity in primary auditory cortex are expressed differently in tinnitus subjects compared to controls matched for age and degree of threshold shift. Subjects in both groups were trained for seven sessions to detect a target sound (a single 40-Hz AM pulse of variable increased amplitude) embedded in a 40-Hz AM sound of 1-s duration, using a carrier frequency of 5 kHz which was in the TFR of the tinnitus group. In agreement with prior results in normal hearing subjects (Bosnyak et al. 2004; Gander et al. 2010b), ASSR amplitude was resistant to remodeling by training in control subjects, suggesting that competitive interactions occurring in primary auditory cortex may have constrained an expansion of the cortical territory representing the trained sound. However, ASSR amplitude increased significantly over training in the tinnitus subjects, suggesting that in tinnitus these constraints were relaxed. A second finding was that ASSR phase (the time delay between the 40-Hz stimulus and response waveforms) did not change with training in the tinnitus group, although this attribute (known to be highly plastic in normal hearing subjects) did so in controls. These results indicate that tinnitus-related activity occurring in the TFR of primary auditory cortex modifies the outcome of auditory training delivered to this region in subjects experiencing tinnitus. Whether different results are obtained when training is given for sounds below the TFR is not presently known.

### Changes in nonauditory brain regions

Functional and structural imaging studies of humans have been a rich source of information about changes in nonauditory pathways that may be related to tinnitus and its correlated conditions. In considering these studies, it is useful to distinguish between hemodynamic imaging methods (fRMI, PET), that can identify areas of net increased or decreased metabolic activity in brain regions related to tinnitus and measure functional coupling between these regions, and electromagnetic methods, that measure changes in synchronous (phase locked) activity of neurons occurring in and between brain regions possibly related to tinnitus. Previous reviews by Lanting et al. (2009; fMRI and PET), Husain and Schmidt (2014), Vanneste and De Ridder (2012; EEG and MEG), Roberts et al. (2013, both approaches), and Adjamian et al. (2009; both approaches) are updated and extended here with discussion in the sections below. It will be seen that, while interpretation of the results of these two approaches is often a challenge, a broad convergence exists in three major respects. First, in agreement with an earlier (Jastreboff 1990) prescient brain model of tinnitus, the neural changes occurring in humans experiencing tinnitus are not confined to auditory pathways. Second, the brain regions affected are known to support behavioral functions related to attention, emotion, memory, and sensorimotor processes. Third, the human and animal data largely agree on the latter point. However, debate continues on whether neural changes occurring in nonauditory structures relate specifically to tinnitus as opposed to its correlated conditions, including hyperacusis, distress behavior, and hearing loss. Also debated is whether neural changes occurring in nonauditory regions are driven by aberrant neural activity occurring in auditory pathways or whether nonauditory regions are more directly involved in the generation of tinnitus sounds.

#### PET, MRI, and FRMI imaging

The cerebellum is involved in some aspects of tinnitus-related hyperactivity as several studies in humans and animals have reported, although the overall picture is not consistent. PET studies of spontaneous activity (Lockwood et al. 2001; Mirz et al. 1999; Osaki et al. 2005) found increased metabolic activity in the cerebellum of tinnitus patients. Residual inhibition (brief suppression of tinnitus after masking) decreased this activity (Osaki et al. 2005). Maudoux et al. (2012) tested whether fMRI “resting-state” connectivity patterns in auditory network behavior differed between tinnitus patients and normal controls. They found that connectivity in extra-auditory regions, such as basal ganglia (nucleus accumbens), cerebellum, parahippocampal gyrus, right prefrontal cortex, parietal cortex, and sensorimotor areas, was increased in tinnitus subjects. However, sound-evoked responses measured from the cerebellum by fMRI do not appear to differ between tinnitus patients and controls (Lanting et al. 2010; Boyen et al. 2014). Melcher et al. (2013) tested for differences in grey matter (GM) volume in the subcallosal region (the area immediately inferior to the anterior corpus callosum and incorporating the ventromedial prefrontal cortex) between tinnitus and control subjects matched for age, sex, handedness, and sound thresholds to 14 kHz. No differences in GM volume or concentration were found in this region between the two groups. However, subsequent whole-brain analyses found that GM volume in the ventral posterior cingulate cortex (vPCC), dorsomedial prefrontal cortex (dmPFC), and the ventromedial prefrontal cortex (vmPFC, this latter structure in the subcallosal region) correlated negatively with pure tone thresholds >8 kHz in the tinnitus and control groups and in the combined sample. GM volume in the cerebellum was positively correlated with anxiety in the combined sample. These results underscored the importance of controlling for threshold shifts above 8 kHz and attributes associated with tinnitus in interpreting brain data.

MRI has been used to investigate structural differences between individuals with and without tinnitus. Boyen et al. (2013) used voxel-based morphometry to compare GM volume between three groups, hearing impaired people with tinnitus, hearing impaired people without tinnitus, and normal hearing controls with each group of similar age. This design allowed one to disentangle the GM differences related to hearing loss and tinnitus, respectively. Relative to the controls, the two patient groups had GM increases in the superior and middle temporal gyri, and decreases in the superior frontal gyrus, occipital lobe and hypothalamus. In agreement with Melcher et al. (2013), no significant GM differences were found between the two patient groups. These results suggest that hearing loss may have been the contributing factor in the GM changes. Subsequent region-of-interest (ROI) analyses of all cortical (Brodmann) areas, the cerebellum, and the subcortical auditory nuclei, showed a GM increase in the left primary auditory cortex of the tinnitus patients compared to the hearing-impaired people without tinnitus and control groups. These results suggested a specific role of the left primary auditory cortex and the additional involvement of various nonauditory brain structures in tinnitus. Boyen et al. (2013) did not draw conclusions on a potential causal relation between GM differences, hearing loss and tinnitus. The GM increase in the left primary auditory cortex of tinnitus subjects could represent a pre-existing vulnerability to develop tinnitus in response to sensory neural hearing loss or it could be a consequence of increased ongoing neural activity presumed to underlie tinnitus (Husain et al. 2011). In a subsequent study of functional changes, Boyen et al. (2014) measured cortical and sub-cortical sound-evoked BOLD responses in 34 hearing-impaired chronic tinnitus patients and 19 hearing level-matched controls and found no differences between both groups in terms of the magnitude and lateralization of the sound-evoked responses, except for the left medial geniculate body and right cochlear nucleus where activation levels were elevated in the tinnitus subjects. They also observed significantly reduced functional connectivity between the inferior colliculi and the auditory cortices in tinnitus patients compared to controls. This suggested to them a failure of thalamic gating in the development of tinnitus.

**Fig 3:**
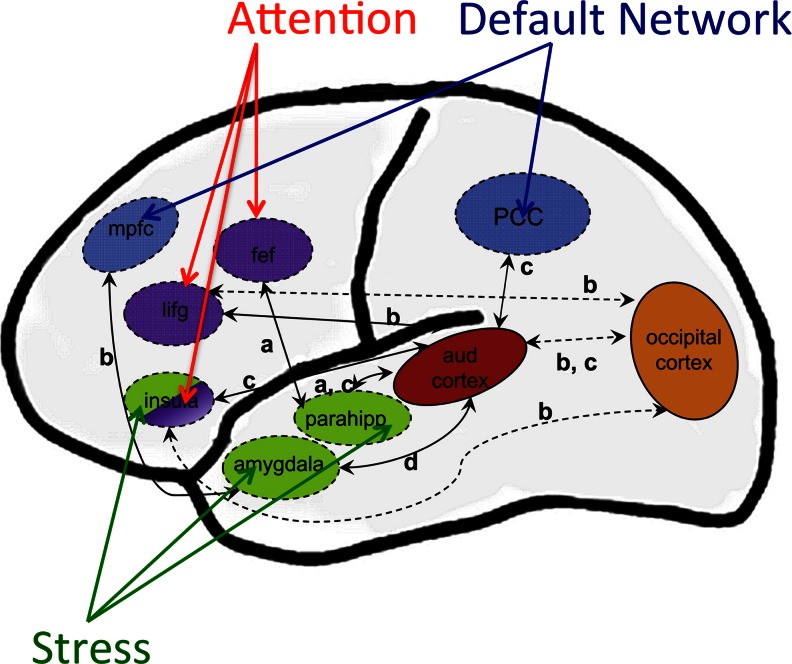
Summary of main results of resting-state functional connectivity studies in tinnitus. The major networks highlighted are default-mode network (DMN, *blue*), limbic network involved in stress (*green*), auditory network (*red*), the visual network (*orange*), several attention networks (specifically the dorsal attention network and the executive control of attention, *purple*). Positive correlations between regions that are stronger in tinnitus patients than controls are shown in *solid lines*; negative correlations are shown as *dashed lines*. Connections are labeled with *letters* representing the studies in which they were reported: *a* Schmidt et al. (2013). *b* Burton et al. (2012). *c* Maudoux et al. (2012). *d* Kim et al. (2012). *PCC* posterior cingulate cortex;* mpfc* medial prefrontal cortex;* lifg* left inferior frontal gyrus;* parahipp* parahippocampus;* aud cortex* auditory cortex;* fef* frontal eye fields. Modified from Husain and Schmidt (2014)

More global changes in resting state functional connectivity in tinnitus have recently been summarized by Husain and Schmidt (2014). Evidence they review suggests that several resting-state brain networks identified in healthy individuals are modified in tinnitus, including the default mode network, auditory attention network, and functionally coupled regions in the limbic system. The principal brain structures involved in these networks, and how connectivity between structures in the different networks is modified in tinnitus, are summarized in Fig. 3 (adapted from Husain and Schmidt 2014). Functional couplings among structures in these networks that are stronger in tinnitus subjects than controls are shown in solid lines. Negative correlations indicate inverse functional coupling between regions; these mostly involve occipital visual areas with auditory and frontal attention systems. Evidence discussed by Husain and Schmidt (2014) suggests that tinnitus is associated with consistent modifications to these networks, including in particular greater connectivity between limbic areas and cortical networks not traditionally involved with emotion processing, and increased connectivity between attention and auditory processing brain regions.

Studies using PET are of interest because this method is in principle sensitive to metabolic demands arising from increased SFR believed to be involved in tinnitus. Furthermore, it is a silent method in contrast to fMRI. Arnold et al. (1996) using [18 F]-fluoro-deoxyglucose positron emission tomography (FDG-PET) reported increased resting metabolic activity in primary auditory cortex for a group of 10 out of 11 patients with chronic tinnitus compared to 14 controls. Mirz et al. (1999) subtracted PET images of regional cerebral blood flow (^15^O-labeled water) obtained when tinnitus was suppressed by masking or lidocaine from PET images obtained during baseline scans where tinnitus was experienced. They found a larger difference in neuronal activity between unmasked and masked conditions in the tinnitus patients compared to the controls. The increased neuronal activity associated with tinnitus occurred predominantly in the right hemisphere with significant foci in the middle frontal and middle temporal gyri, in addition to lateral and mesial posterior sites. The results were consistent with the hypothesis that the sensation of tinnitus is associated with activity in cortical regions functionally linked to areas subserving attention, emotion and memory. Group analysis of PET data (Plewnia et al. 2007) showed tinnitus-related increases of regional cerebral blood flow in the left middle and inferior-temporal cortex as well as right temporoparietal cortex and posterior cingulum, when compared to activity following intravenous lidocaine that induced a suppression of tinnitus. The group data showed no significant tinnitus-related hyperactivity (activity blocked by lidocaine) in the primary auditory cortex (BA 41). The regions affected by lidocaine were in the human equivalent of the parabelt areas of auditory cortex. Andersson et al. (2000) found after lidocaine infusion that tinnitus was associated with increased regional cerebral blood flow (rCBF) in the left parieto-temporal auditory cortex, including the primary and secondary auditory cortex with a focus in the parietal cortex (BA 39, 41, 42, 21, and 22). Activations were also found in right frontal paralimbic areas (BA 47, 49, and 15). A cautionary finding in interpreting these data was provided recently by Geven et al. (2014) who used FDG-PET to measure brain metabolism in 20 tinnitus patients and to compare their results to those in 19 control subjects without tinnitus. The two groups were matched for age (50.1 ± 10 years) and had audiograms showing similar but not identical mild threshold shifts commencing above 2 kHz and rising to about 50 dB at 8 kHz. In contrast to their expectation, no hyperactivity was found when comparing the tinnitus and control groups. Nevertheless, the activity in the left primary auditory cortex was higher than in the right primary auditory cortex, but this asymmetry was present in both tinnitus patients and control subjects. In contrast, the lateralization in secondary auditory cortex was the opposite, with higher activation in the right hemisphere, again in both groups. These data show that hemisphere asymmetries in the metabolic resting activity of the auditory cortex are present, but these are not associated with tinnitus and are instead a normal characteristic of the brain, at least for individuals of this age and hearing loss.

#### EEG and MEG

Largely due to the extensive analyses of resting brain EEG/MEG in tinnitus patients by De Ridder’s group (originally in Antwerp, Belgium) and Weisz and colleagues (originally in Konstanz, Germany), there are now several reports of resting-state oscillatory brain changes recorded electromagnetically in tinnitus patients compared to controls under baseline conditions. Table 2 compiles findings in spontaneous EEG and MEG, i.e., power changes in various frequency bands, which reflect changes in neural synchrony in tinnitus patients. The findings include deceased auditory alpha (10–14 Hz) (Weisz et al. 2005), increased slow-wave delta activity (1.5-4 Hz) (Weisz et al. 2005), increased gamma activity (40–60 Hz) coupled to slow oscillations in auditory cortex (Weisz et al. 2007), and increased gamma oscillations that track the laterality of the tinnitus percept (Weisz et al. 2007; Van der Loo et al. 2009). Adjamian et al. (2012) similarly observed increased delta activity in tinnitus but alpha power was not reduced in this study. Oscillatory activity was recorded in 10-s epochs inserted between masking sounds of similar duration which may have altered the tinnitus percept. Lorenz et al. (2009) found that decreases in alpha power in tinnitus subjects were associated with increased gamma power, suggesting that the mechanisms generating alpha may be involved in regulating the balance of excitation and inhibition in cortical regions. Following Llinás et al. (2005), Weisz et al. (2007) proposed that slow wave oscillations in tinnitus reflect hyperpolarization of thalamic nuclei consequent on deafferentation, which in turn disinhibits thalamocortical oscillations in the 40-Hz range giving rise or contributing to synchronous activity underlying the tinnitus percept. Increased slow wave activity in tinnitus is among the most consistent findings reported in Table 2, whereas the findings for alpha are less consistent. Increased gamma activity during silence in individuals experiencing tinnitus may play the same role in perception as stimulus-induced gamma activity in persons without tinnitus. According to this view, increased gamma activity in tinnitus is a neural code for the phantom percept (Weisz et al. 2005; De Ridder et al. 2011).

**Table 2:** Findings in spontaneous EEG and MEG

Area/rhythm	Delta	Theta	Alpha	Beta	Gamma
AC-contra	⇑^a,b,g^		⇓^a^ ≈ ^g^		⇑^a,c,e^ ≈ ^g^
AC-ipsi					⇑^c^
AC-left (bilateral tinnitus)	⇑^d^	⇑^d^	⇑^d^	⇑^d^	⇑^d^
Anterior cingulate	⇑^d^	⇑^d^	⇑^d^	⇑^d^	⇑^d,f^
Posterior cingulate			⇓^f^		
(Posterior) insula	⇑^d^	⇑^d^	⇑^d^	⇑^d^	⇑^d,f^
Parahippocampal area	⇑^d^	⇑^d^	⇑^d^	⇑^d^	⇑^d,f^
Amygdala					⇑^f^
Precuneus			⇓^f^		
Dorso-lateral prefrontal cortex			⇓^f^		

^a^Weisz et al. (2005); ^b^Weisz et al. (2007); ^c^Ashton et al. (2007); ^d^Moazami-Goudarzi et al. (2010); ^e^Van der Loo et al. (2009); ^f^Vanneste et al. (2010); ^g^Adjamian et al. (2012)

Recent findings by Sedley et al. (2012) have posed a challenge to this view. These researchers used MEG to record oscillatory activity in a group of 17 patients with chronic tinnitus before and after maskers intended to induce some degree of RI or the opposite, called residual excitation. The masking sounds produced RI in most subjects (*n* = 15) but residual excitation (a post-masking increase in tinnitus) in other subjects (*n* = 5). In a whole-brain analysis using beamforming methods, 30 clusters of significant power changes between tinnitus high and tinnitus low were detected within subjects, which were heterogeneous in terms of their frequency ranges and in terms of the direction of power change for a given cortical area between subjects. A wide region of the brain was covered by the clusters, but the most consistent clusters showing tinnitus high/low differences (besides the auditory cortex discussed below) were in the cerebellum, anterior temporal lobe, posterior cingulate cortex, ventromedial prefrontal cortex, medial occipital lobe, and anterior cingulate cortex. More consistent changes related to tinnitus changes were found in the auditory cortex. During RI, Sedley et al. found increases in gamma band oscillations relative to a control masker during epochs in which tinnitus was increased in qualitative agreement with the results of Weisz et al. (2007), but, during residual excitation, epochs of tinnitus increase compared to control were associated with decreased gamma. On the basis of this finding, it was concluded that increased gamma could not be a simple neural code for tinnitus. Changes in delta and theta power were also observed with fluctuations in tinnitus, but only in association with RI where power increases were positively correlated with tinnitus intensity. This correlation was interpreted as reflecting increased thalamocortical input to the cortex when tinnitus was experienced, or as Boyen et al. (2014) expressed it, a failure of thalamic gating.

Other studies have examined communication among brain regions in subjects with tinnitus compared to control conditions. Schlee et al. (2008) reported increased phase locking of oscillatory responses among the frontoparietal, temporal, and cingulate cortices in tinnitus patients compared to controls, with greater involvement of frontal and parietal regions in the longer term compared to acute cases of tinnitus (Schlee et al. 2009a). These connectivities were expressed predominantly in the alpha (9–12 Hz) and gamma (48–54 Hz) bands (Schlee et al. 2009a). Stronger top–down inflow to temporal cortex from prefrontal, orbitofrontal, and parieto-occipital regions was also found to correlate positively with tinnitus distress (Schlee et al. 2009b). Notwithstanding that inverse modeling of EEG and MEG sources is subject to limitations (see Palva and Palva 2012 for a discussion), low-resolution electromagnetic tomography (LORETA) and similar methods applied to EEG data have been used to describe resting-state oscillatory activities in coarsely imaged brain regions in individuals with tinnitus compared to various control conditions (Schlee et al. 2008; Vanneste et al. 2010). Results reviewed by Vanneste and De Ridder (2012) point to tinnitus-related oscillatory changes occurring in several regions including the auditory cortex, the dorsal anterior and posterior cingulate cortex, dorsolateral prefrontal cortex, regions of frontal cortex, and the parahippocampus. While the functional roles of oscillatory activities in these regions and their precise localizations are not well established, they could relate to different aspects of tinnitus including the retrieval of its encoding from memory, its attended conscious experience, or distress behavior associated with a persistent annoying phantom sound (Vanneste and De Ridder 2012). Of the brain areas showing changes in oscillatory activity in tinnitus, regions of the auditory cortex (Paltoglou et al. 2009), anterior cingulate (Sadaghiani et al. 2009), and prefrontal cortex (Voisin et al. 2006) are activated when normal hearing subjects attend to anticipated sound stimuli on cognitive tasks.

### Functional role of nonauditory changes

Converging animal and human evidence reviewed above supports the view that structure and function are modified in nonauditory brain regions in tinnitus, and that the brain regions most consistently affected are those known from neuroscience studies of healthy subjects to support attention (a network involving prefrontal cortex, the cingulate gyrus, and striatum), emotion (the amygdala), and memory (hippocampal and parahippocampal regions). At this time, three viewpoints can be distinguished regarding the role of nonauditory regions in the development and experience of tinnitus.

The first viewpoint proposes that functional and structural changes in nonauditory regions are driven by tinnitus-related neural activity occurring in central auditory pathways that is necessary and sufficient for the experience of tinnitus. While the nonauditory changes are therefore not crucial for tinnitus perception, they are responsible for its associated consequences such as persistent activity in auditory attention networks (Roberts et al. 2013), upregulation of somatosensory inputs to auditory pathways (Zeng et al. 2012), and correlated distress behavior, including anxiety, sleeplessness, and depression (Kraus and Canlon 2012). This viewpoint allows that, while nonauditory involvement is not essential for the perception of tinnitus, changes taking place in nonauditory structures may modulate tinnitus severity or its persistence and the changes associated with it.

Two other viewpoints accord an active role for nonauditory structures in the generation of tinnitus. These views were motivated initially by an attempt to understand involvement of nonauditory regions in tinnitus revealed by human functional imaging data. They have also attracted interest by the necessity to explain the occurrence of tinnitus in individuals not presenting audiometric thresholds shifts (TIN–TS) and of individuals with threshold shifts but no tinnitus (TS–TIN; for examples of both in group data, see Roberts et al. 2008). The first of the two viewpoints was advanced by De Ridder et al. (2011) on the basis of evidence much of which has been discussed in the sections above. According to this view, deafferentation of auditory pathways by cochlear damage results in hyperpolarization of thalamic auditory nuclei and high-frequency, synchronized gamma band neural activity in auditory cortex (thalamocortical dysrhythmia). However, this activity becomes a conscious percept only if it is connected to brain networks responsible for self-awareness and assigning salience to perceptual objects. In turn, interactions of the salience network through subcallosal regions and the amygdala modulate the reticular nucleus of the thalamus, thereby further contributing to thalamocortical dysrhythmia. Through learning mechanisms activated by this process, the tinnitus percept becomes associated with network activity in brain areas involved in emotion and memory (the parahippocampal area, cingulate cortex, insula and amygdala). This model aligns with evidence for changes in nonauditory brain regions in tinnitus and opens the possibility that dissociations between the occurrence of tinnitus and audiometric hearing loss may reflect not cochlear factors but individual differences in access to brain networks responsible for conscious awareness.

An alternative viewpoint advanced at about the same time (Rauschecker et al. 2010) similarly proposed that cochlear damage, while a triggering factor, was not sufficient for the perception of tinnitus. According to this model, hyperactivity in auditory pathways consequent on hearing impairment loss is under normal circumstances cancelled out at the level of the thalamus by an inhibitory feedback loop (noise cancellation system) originating in the ventromedial prefrontal cortex and nucleus accumbens. These structures are part of an established canonical circuit in the subcallosal ventral striatum and are proposed here to identify the presence of unwanted neural activity in auditory pathways (see also Boyen et al. 2014). The unwanted neural activity (tinnitus signal) is fed back to the reticular nucleus of the thalamus, which removes the signal from input to the auditory cortex. According to this model, disparities between the presence of hearing loss and tinnitus depend on individual differences in the effectiveness of the noise cancellation system supported from nonauditory regions. The model was originally motivated by structural imaging results revealing a structural loss of GM in the nucleus accumbens in individuals reporting tinnitus compared to controls (Muhlau et al. 2006) which was subsequently proposed to be a neural signature for defective gating. In support of the model, Leaver et al. (2011) reported further results revealing functional and structural correlates of chronic tinnitus in limbic and auditory regions of the human brain. In particular, in tinnitus patients, the NAc exhibited hyperactivity specifically for stimuli matched to each patient’s tinnitus frequency. Corresponding anatomical differences were identified in the vmPFC which is strongly connected to the ventral striatum. Within the auditory cortex, Leaver et al. (2011) noted hyperactivity in medial Heschl’s gyrus (mHG), which was restricted to tinnitus frequency-matched stimuli and was also positively correlated with tinnitus-related limbic abnormalities.

The noise cancellation model has attracted interest for its novelty and the active role in the gating of tinnitus accorded to it by nonauditory regions. Reservations have been reported, however. Reports of tinnitus-related structural differences in the subcallosal brain region that gave rise to the hypothesis have not been corroborated (Adjamian et al. 2014). Landgrebe et al. (2009) found no differences between tinnitus and control subject groups in the subcallosal brain. Husain et al. (2011) compared tinnitus and non-tinnitus control subjects with hearing loss, as well as control subjects with clinically normal hearing, and found no differences related to tinnitus in subcallosal brain. In a study described earlier, Melcher et al. (2013) tested for differences in GM volume and concentration in the subcallosal region between tinnitus and control subjects matched for age, sex, handedness, and sound thresholds to 14 kHz. In contrast to Muhlau et al. (2006) and Leaver et al. (2011), no differences were found in GM volume or concentration between the two groups. However, GM volume in the vmPFC (also in the ventral posterior cingulate cortex and dorsomedial prefrontal cortex) correlated negatively with pure tone thresholds between 8 and 14 kHz in the tinnitus and control groups separately and in the combined sample. The results called attention to the importance of controlling for high-frequency threshold shifts in interpreting brain data, which was not done in the earlier studies. It is also not clear how unwanted neural activity occurring in auditory pathways is identified by nonauditory structures (a process that may require significant spectral-temporal processing of sound). Taking a different approach, Roberts et al. (2013) described how predictive filtering by primary auditory cortex could detect the presence of tinnitus-related neural activity occurring in auditory pathways. However, the outcome of detection in this model is not noise cancellation but activation of neuromodulatory attention systems in the forebrain that in normal hearing support construction by the cortex of a more accurate representation of the auditory scene. In tinnitus, this process fails, owing to the disparity that exists between the aberrant tinnitus-related afferent neural activity the auditory cortex predicts it should be receiving but which is not delivered to the brain by the damaged cochlea. This model forecasts neural changes in nonauditory brain regions but does not (and was not intended to) account for dissociations between tinnitus and threshold shift (TIN–TS or TS–TIN). If explanation of these cases in terms of nonauditory processing seems less than satisfying, how are they to be explained otherwise?

## Cochlear pathology revisited: animals and humans

The models discussed above can be classified as extracochlear models, in the sense that central factors and not changes in the ear are invoked to explain cases of TIN-TS and TS-TIN. Recent human and animal studies have, however, rekindled interest in the role of cochlear pathology in the generation of tinnitus.

Threshold shifts in the audiogram associated with aging or noise exposure reflect reduced input from the cochlea following damage to the cochlear transduction mechanism (OHCs and IHCs and the structures supporting them), or injury to low threshold high spontaneous rate ANFs in the cochlear nerve (these ANFs conveying information about just-detectable sounds to the central auditory system), or both factors. During the course of aging, the threshold shifts up to ~40 dB at 4 kHz commencing at ~50 % of the lifespan (mouse, but resembling humans) and are closely paralleled by OHC loss, suggesting damage to the transduction mechanism as the principal cause (Sergeyenko et al. 2013). One explanation for cases of TS–TIN could thus be that damage to the transduction mechanism affecting auditory thresholds is not yet severe enough in these cases to release the cascade of neural changes in central pathways that underlie tinnitus (Singer et al. 2013). Consistent with this hypothesis, Roberts et al. (2008) found that, when tinnitus sufferers over the age of 50 years were compared to age-matched controls without tinnitus, threshold shifts were about 12 dB greater in the TIN + TS group over the frequency range where tinnitus percepts lie, suggesting more cochlear damage in this group. However, other data reported by Roberts et al. (2008) refute this hypothesis. The threshold shifts between 4 and 10 kHz seen in the age-matched controls over age 50 (45.7 dB) were 32.8 dB greater than those seen in a tinnitus group aged less than 50 years (14.9 dB) who had clinically normal audiograms (thresholds <20 dB HL to 8 kHz), but the former group did not have tinnitus despite evidence for greater cochlear impairment.

This line of reasoning from human data shifts the spotlight to ANFs, particularly to high threshold, low spontaneous rate ANFs synapsing on IHCs with high-frequency tuning which have been implicated in tinnitus (see “Tinnitus and neural plasticity”). Animal data show that ribbon synapses on these ANFs are more vulnerable to noise damage than those on low threshold ANFs (Furman et al. 2013; Kujawa and Liberman 2009) and that damage to them does not recover with time (Kujawa and Liberman 2009; Lin et al. 2011). When input–output (loudness growth) functions are measured in an animal model after noise trauma, the output of the cochlea measured by ABR wave I is reduced at high sound intensities reliant on these fibers, whereas wave IV is enhanced (Kujawa and Liberman 2009) revealing increased central gain. Human data fit this pattern remarkably well, showing reduced wave I amplitude (Gu et al. 2012; Schaette and McAlpine 2011) and increased wave V amplitude (Gu et al. 2012) as well as steeper input–output functions measured behaviorally in individuals with tinnitus and normal thresholds (<15 dB HL) compared to age- and hearing level-matched controls (Hébert et al. 2013).

Bharadwaj et al. (2014) and others have described a reduction in the number of ANFs responding to supra-threshold sound as “cochlear neuropathy”. The presence of tinnitus itself, as well as abnormal neural and behavioral responses to supra-threshold sounds, could indicate the presence of this condition. Cochlear neuropathy may underlie other disorders of hearing such as difficulties of hearing in noise where the acoustic background may be sufficient to saturate low threshold ANFs, removing their contribution to temporal coding of the speech envelope leaving only that of damaged high threshold ANFs. This point of view places tinnitus in the context of other hearing disorders where temporal processing may be similarly impaired. Parenthetically, this view applied to tinnitus suggests the hypothesis that older persons with threshold shift but not tinnitus (TS–TIN over age 50) may owe their threshold shift primarily to changes in the transduction mechanism associated with aging. Individuals with tinnitus closely matched in age to this group may have similar age-related changes in the transduction mechanism giving their threshold shift, but may in addition experience cochlear neuropathy owing to noise exposure in their personal histories giving their tinnitus.

The question of how far cochlear factors may go toward explaining TIN–TS and TS–TIN has yet to be determined. The relationship of cochlear factors to tinnitus may involve additional mechanisms, since cochlear ablation up to 8 weeks post-trauma but not later abolishes hyperactivity in the inferior colliculus (Mulders and Robertson (2009, 2011), suggesting the dependence of changes in central gain on cochlear input. The line of reasoning described here shifts the focus of research and intervention in tinnitus to hearing restoration and to the early detection and prevention of hearing injuries. It also calls attention to the study of the relationship of tinnitus to other disorders of hearing where temporal processing may be impaired by forms of cochlear neuropathy. Understanding this relationship may require assessment of hearing in control subjects under different levels of background noise including silence, to preclude masking of an undetected tinnitus by environmental sounds.

## Summary, conclusions, and limitations

In this article, we have reviewed findings from animal models of tinnitus and from studies of human tinnitus sufferers regarding the neural mechanisms that underlie this disorder. Animal studies concur that, following tinnitus-inducing procedures (salicylate and noise trauma), neural gain is increased in subcortical auditory structures and in the auditory cortex, as expressed by increased driven neural responses in these structures and by behavioral measures. Increases in SFR are also observed after noise trauma in several central auditory nuclei, and occur in this case against a background of reduced activity in ANFs, whereas, following salicylate, changes in SFR are more variable across auditory nuclei and are associated with increased SFR in ANFs. This difference between the effects of noise trauma and salicylate on SFR could be secondary to different levels of activity in the cochlear nerve following these procedures, or to mechanisms that modulate SFR different from changes in central gain. In this regard, computational models suggest that changes in central gain may be sufficient to increase SFR in central auditory pathways, which could be the neural basis for tinnitus. However, caution is needed, because changes in SFR and central gain are inconsistently correlated in the animal data. Other neural mechanisms that may underlie tinnitus (such as increased neural synchrony, tonotopic map reorganization, or hyperpolarization of thalamic nuclei consequent on deafferentation) have received less investigation in animal studies. A challenge for animal models going forward is to rule out impaired temporal processing or hyperacusis as the source of neural and behavioral changes seen in these studies, separately from tinnitus. Within the limits of this challenge, animal studies have provided evidence for the centralization of tinnitus over time, and have revealed neuroplastic mechanisms that may contribute to the development of tinnitus percepts.

An advantage of human studies is that the presence or absence of tinnitus can be reported verbally and verified by psychoacoustic measurements. Electromagnetic imaging studies of human tinnitus sufferers have revealed changes in synchronous neural population activity in the auditory cortex in different frequency bands, the most consistent being increased slow wave activity (delta and theta, <4 Hz) and increased gamma oscillations (>40 Hz) from the region of the auditory cortex with several reports of reduced alpha activity (8–12 Hz) as well. Increased delta may reflect deafferentation of thalamocortical projections leading to increased gamma oscillations in local networks consequent on disinhibition of cortical neurons. Reduced alpha is thought to signal desynchronization of longer-range network activity in the auditory cortex following reduced inhibition in (or the deployment of attention to) this region. These results regarding oscillatory responses are consistent with animal studies that point to aberrant neural synchrony as a possible mechanism for tinnitus. Because the findings are obtained from resting neuromagnetic baselines, their spectral (tonotopic) signatures cannot be determined, but data from cats subjected to noise trauma show increased synchronous activity confined to tonotopic frequency regions affected by hearing loss, which is also where human tinnitus percepts lie. Functional imaging of tinnitus in humans has further revealed increased activity in nonauditory brain networks known to be involved in consciousness, memory, and emotion, with the limited animal data currently available corroborating these changes when physiological measurements extend to these regions. Several functional roles have been proposed for nonauditory regions, including most notably gating access to dorsal prefrontal structures responsible for conscious perception, which could explain dynamic fluctuations of tinnitus awareness with task demands. Nonauditory mechanisms involved in tinnitus may also account for dissociations between the co-occurrence of tinnitus and threshold shift, but another possibility raised by recent animal studies and supported findings in humans points to undetected cochlear neuropathy in these cases.

Animal models permit invasive experimentation that cannot be conducted in humans, and results obtained from them can guide human investigations. A good example of how the two approaches have been complementary are studies of human tinnitus patients revealing increased gain in ABRs recorded for suprathreshold sounds and steepened behavioral loudness growth functions compared to controls, both results confirming expectations based on animal data. Going in the other direction, evidence for involvement of nonauditory regions in tinnitus obtained from human functional imaging has been corroborated by recent animal experiments which have extended their observations to these regions. Animal and human studies are also converging to suggest a role for trophic mechanisms in establishing correlates of tinnitus, including structural and functional neuroplastic changes that occur in the brain following damage to the cochlea. Looking ahead, animal and human studies are likely to provide important insight into several emerging topics, including the role of auditory and nonauditory network behavior in tinnitus, contributions from neural plasticity, and factors that modulate the experience of tinnitus as well as its generation.

Because studies of humans and animals necessarily involve different measurement techniques, a continuing challenge will be to understand how measurements at different levels of function relate to one another, given the constraints inherent in any single method. Animal recordings related to tinnitus reflect single- or multi-unit spontaneous firing rates, spontaneous neural synchrony based on pair-wise cross-correlations, and stimulus-evoked firing rate or LFP amplitudes and the tonotopic maps constructed therefrom. In contrast, human data are always population data reflecting activity of at least 10^5^ neurons. fMRI does not require synchronously driven activity, whereas EEG/MEG derived data do. Spontaneous activity can only be obtained by using PET (because it is a silent technique) that reflects the metabolic demand of the neurons. The most relevant measure in animals relating to PET measures in humans is the 2-DG one, and here the recording is likely biased toward neurons whose activity is detectable (i.e., those with not too low firing rates). The macroscopic stimulus-driven BOLD responses and AEP/AEF measure only central gain differences between tinnitus groups and controls, and therefore may relate more to hyperacusis than to tinnitus (Gu et al. 2010). These responses can be favourably compared to their mesoscopic equivalent in animal research, the LFP. The power of the standard range of brain rhythms (2–60 Hz) in EEG/MEG reflects both the number of participating neurons and the spatial orientation and the size of their dipole moments. Neural synchrony comes in here in the size of the equivalent dipole moments for a large group of neurons that allow these rhythms to be detected on the scalp.

The problem of relating measurements at different levels is highlighted by the observation that evidence linking gamma-band oscillations to spike pair correlations is not completely consistent. Cardin et al. (2009) found that light-driven (optogenetic) activation of fast-spiking interneurons at varied frequencies (8–200 Hz) in barrel cortex selectively amplified gamma oscillations, while activation of pyramidal neurons amplified only lower frequency oscillations, revealing a cell-type-specific double dissociation. Given that the cortical spike–spike correlations measured in the animal models of tinnitus were between putative pyramidal cells, it is not straightforward to associate the increased correlation strength for auditory cortical pyramidal cells in animal models with increased power of gamma-band oscillations in humans with tinnitus. However, other studies make the case for pyramidal cell pair correlations and gamma power in the cortices. Denker et al. (2011) presented experimental evidence to reconcile the notions of synchrony at the level of cell spiking and at the mesoscopic LFP scale in motor cortex. They demonstrated that in time intervals of significant pyramidal cell spike synchrony that could not be explained on the basis of firing rates, coincident spikes were better phase locked to the LFP than predicted by the locking of the individual spikes. This suggested that precise spike synchrony constitutes a major temporally and spatially organized component of the LFP. Jia et al. (2013) showed that elevated gamma power is associated with stronger pyramidal cell spike–spike correlation both within and between (visual) cortical areas. Lee and Lisberger (2013) found that the strength of the spike-field coherence of a neuron in the gamma-band frequency range is related to the size of the pyramidal cell pair correlations. These data suggest that cortical rhythm changes are mostly in agreement with increased neural synchrony at the level of the auditory cortex.

Looking ahead to future animal studies, physiological and optogenetic methods offer the prospect of investigating the fine detail of thalamocortical dynamics in tinnitus as well as the role neuromodulators and forms of plasticity that are only beginning to receive attention in the tinnitus literature. Future animal studies may also provide insight into the intracellular and molecular mechanisms that are involved in tinnitus as well as in the processing of sound by the intact brain. Research into how results from these different methods are related by their underlying mechanisms will be needed to integrate the findings and to extrapolate them to humans where different methods of study must be used.

## Abbreviations

2-DG: 2-deoxyglucose
ABR: Auditory brain stem response
AC: Auditory cortex
AI: Primary auditory cortex
AII: Secondary (nonprimary) auditory cortex
ANF: Auditory nerve fiber
ASR: Acoustic startle response
ASSR: Auditory steady-state-response
BDNF: Brain derived neurotrophic factor
BLA: Basolateral amygdala
CAP: Compound action potential
CF: Center frequency
Cg: Cingulate gyrus
CRH: Corticotrophin releasing hormone
DCN: Dorsal cochlear nucleus
dmPFC: Dorsomedial prefrontal cortex
DPOAEs: Distortion product otoacoustic emissions
EEG: Electroencephalography
FMRI: Functional magnetic resonance imaging
GM: Grey matter
GR: Glucocorticoid receptor
HC: Hippocampus
HPA: Hypothalamic pituitary axis
IC: Inferior colliculus
IHC: Inner hair cell
LA: Lateral amygdala
LFP: Local field potential
MEG: Magnetoencephalography
MR: Mineralocorticoid receptor
NMDA: N-methyl-D-aspartate receptor
OHC: Outer hair cell
PFL: Paraflocculus of the cerebellum
PET: Positron emission tomography
PPI: Pre-pulse inhibition
RI: Residual inhibition
SBC: Spherical bushy cells
SFR: Spontaneous firing rate
SPL: Sound pressure level
STDP: Spike timing-dependent plasticity
TFR: Tinnitus frequency region
VCN: Ventral cochlear nucleus
VOT: Voice onset time
vmPFC: Ventromedial prefrontal cortex
vPCC: Ventral posterior cingulate cortex
